# Guideline on allergen immunotherapy in IgE-mediated allergic diseases: S2K Guideline of the German Society of Allergology and Clinical Immunology (DGAKI), Society of Pediatric Allergology and Environmental Medicine (GPA), Medical Association of German Allergologists (AeDA), Austrian Society of Allergology and Immunology (ÖGAI), Swiss Society for Allergology and Immunology (SSAI), German Dermatological Society (DDG), German Society of Oto-Rhino-Laryngology, Head and Neck Surgery (DGHNO-KHC), German Society of Pediatrics and Adolescent Medicine (DGKJ), Society of Pediatric Pulmonology (GPP), German Respiratory Society (DGP), German Professional Association of Otolaryngologists (BVHNO), German Association of Paediatric and Adolescent Care Specialists (BVKJ), Federal Association of Pneumologists, Sleep and Respiratory Physicians (BdP), Professional Association of German Dermatologists (BVDD)

**DOI:** 10.5414/ALX02331E

**Published:** 2022-09-06

**Authors:** Oliver Pfaar, Tobias Ankermann, Matthias Augustin, Petra Bubel, Sebastian Böing, Randolf Brehler, Peter A. Eng, Peter J. Fischer, Michael Gerstlauer, Eckard Hamelmann, Thilo Jakob, Jörg Kleine-Tebbe, Matthias Volkmar Kopp, Susanne Lau, Norbert Mülleneisen, Christoph Müller, Katja Nemat, Wolfgang Pfützner, Joachim Saloga, Klaus Strömer, Peter Schmid-Grendelmeier, Antje Schuster, Gunter Johannes Sturm, Christian Taube, Zsolt Szépfalusi, Christian Vogelberg, Martin Wagenmann, Wolfgang Wehrmann, Thomas Werfel, Stefan Wöhrl, Margitta Worm, Bettina Wedi, Commenting participation and process support:, Susanne Kaul, Vera Mahler, Anja Schwalfenberg

**Affiliations:** 1 Department of Otorhinolaryngology, Head and Neck Surgery, Section of Rhinology and Allergy, University Hospital Marburg, Philipps-Universität Marburg, Marburg,; 2 Department of Pediatrics, Städtisches Krankenhaus Kiel, Kiel,; 3 Institute for Health Services Research in Dermatology and Nursing, University Medical Center Hamburg, Hamburg,; 4 ENT practice Dr. Bubel, Eisleben,; 5 Specialized Practice in Pneumology, Allergology and Sleep Medicine, Düsseldorf/Meerbusch,; 6 Department of Dermatology, University Hospital Münster, Münster, Germany,; 7 Section of Pediatric Pulmonology and Allergy Children’s Hospital, Aarau, Switzerland,; 8 Practice for Pediatric and Adolescent Medicine m.S. Allergology and Pediatric Pneumology, Schwäbisch Gmünd,; 9 Paediatric Pulmonology and Allergology, University Medical Center Augsburg, Augsburg,; 10 Department of Paediatrics, Children‘s Center Bethel, University Bielefeld, Bielefeld,; 11 Department of Dermatology and Allergology, University Medical Center, Justus Liebig University Gießen, Gießen,; 12 Allergy & Asthma Center Westend, Outpatient Clinic & Research Center, Berlin, Germany,; 13 Department of Paediatrics, Inselspital, Bern University Hospital, University of Bern, Bern, Switzerland,; 14 Charité Universitätsmedizin Berlin, Pediatric Respiratory Medicine, Immunology and Critical Care Medicine, Berlin,; 15 Asthma-Allergiezentrum Leverkusen,; 16 Medical Center – University of Freiburg, Center for Pediatrics, Department of General Pediatrics, Adolescent Medicine and Neonatology, Freiburg,; 17 Pediatric Pneumology and Allergology (medical practice), Children’s Center Dresden-Friedrichstadt (Kid), Dresden,; 18 University AllergyCenter Dresden, University Hospital Dresden (UKD), Dresden,; 19 Department of Dermatology and Allergology, University Clinic, Philipps-Universität Marburg, Marburg,; 20 Department of Dermatology, University Medical Center, Johannes Gutenberg-University, Mainz,; 21 Private Office Dermatology, Ahaus, Germany,; 22 Allergy Unit, Dept. Of Dermatology, University Hospital of Zurich, Zurich, Switzerland,; 23 Department of Pediatrics, Düsseldorf University Hospital, Düsseldorf, Germany,; 24 Department of Dermatology and Venerology, Medical University of Graz, Allergy Outpatient Clinic Reumannplatz, Vienna, Austria,; 25 Department of Pulmonary Medicine, University Hospital Essen – Ruhrlandklinik, Essen, Germany,; 26 Department of Pediatrics and Adolescent Medicine, Division of Pediatric Pulmonology, Allergology and Endocrinology, Comprehensive Center Pediatrics, Medical University of Vienna, Vienna, Austria,; 27 Department of Pediatric Pneumology and Allergology, University Hospital Carl Gustav Carus Dresden, Technical, University Dresden, Dresden,; 28 Department of Otorhinolaryngology (HNO-Klinik), Düsseldorf University Hospital (UKD), Düsseldorf,; 29 MVZ Dermatology and Dermatological Surgery Münster, Münster,; 30 Department of Dermatology & Allergy, Comprehensive Allergy Center, Hannover Medical School, Hannover, Germany,; 31 Floridsdorf Allergy Center (FAZ), Vienna, Austria,; 32 Charité – Universitätsmedizin Berlin, corporate member of Freie Universität Berlin and Humboldt-Universität zu Berlin, Department of Dermatology and Allergy, Berlin,; 33 Paul-Ehrlich-Institut, Langen, and; 34 German Allergy and Asthma Association, Mönchengladbach, Germany

**Keywords:** allergen immunotherapy, hyposensitization, guideline, allergen, allergen extract, allergic disease, allergic rhinitis, allergic rhinoconjunctivitis, allergic asthma

## Abstract

Not available

Stage: S2k 

AWMF registry number: 061-004 

Completion: June 30, 2022 

Valid until: June 29, 2027 

ICD-10 numbers: J30.4, J30.1, J30.3, H10.1, H10.8, J45.0, R94.2, T63.4, T 88.6, L50.0, Z51.6, Z91.0 

German version: www.doi.org/10.5414/ALX02331

[Table TableAbbreviations]

## Chapter 1. Guideline development and objectives 

The present guideline on allergen immunotherapy (AIT) was prepared on behalf of and financed by the German Society of Allergy and Clinical Immunology (DGAKI) and replaces the S2 guideline published in 2014 [1]. It has been devised as an S2k guideline in accordance with the standardized procedures of the German “Arbeitsgemeinschaft der Wissenschaftlichen Medizinischen Fachgesellschaften (AWMF)”. A detailed guideline report based on the AWMF procedure (Deutsches Leitlinien-Bewertungsinstrument (DELBI) criteria 1 – 7) can be found on the homepage of the AWMF: https://www.awmf.org/leitlinien/detail/ll/061-004.html. 

In summary, as agreed by the DGAKI board of directors in 2018, the corresponding author was commissioned to coordinate the updating of the guideline, and Bettina Wedi was appointed as co-coordinator during the 1^st^ consensus meeting in 2019. In addition to the members of DGAKI (Oliver Pfaar, Matthias Augustin, Thilo Jakob, Jörg Kleine-Tebbe, Eckard Hamelmann, Susanne Lau, Wolfgang Pfützner, Bettina Wedi, Thomas Werfel, Margitta Worm) representatives of the following organizations were involved in the consensus process: Medical Association of German Allergologists (AeDA) (Randolf Brehler, Norbert Mülleneisen, Katja Nemat, Wolfgang Wehrmann), Society of Pediatric Allergology and Environmental Medicine (GPA) (Tobias Ankermann, Antje Schuster, Christoph Müller), Austrian Society of Allergology and Immunology (ÖGAI) (Gunter Sturm, Zsolt Szépfalusi, Stefan Wöhrl), Swiss Society for Allergology and Immunology (SGAI) (Peter Eng, Peter Schmid-Grendelmeier), German Dermatological Society (DDG) (Joachim Saloga), German Society of Oto-Rhino-Laryngology, Head and Neck Surgery (DGHNO-KHC) (Martin Wagenmann), German Society of Pediatrics and Adolescent Medicine (DGKJ) (Michael Gerstlauer, Christian Vogelberg), Society of Pediatric Pulmonology (GPP) (Matthias Volkmar Kopp), German Respiratory Society (DGP) (Christian Taube), German Professional Association of Otolaryngologists (BVHNO) (Petra Bubel), German Association of Paediatric and Adolescent Care Specialists (BVKJ) (Peter Fischer), Federal Association of Pneumologists, Sleep and Respiratory Physicians (BdP) (Sebastian Böing), Professional Association of German Dermatologists (BVDD) (Klaus Strömer) were taking part in the process of the guideline update. The German regulatory authority, Paul-Ehrlich-Institut (PEI) (Susanne Kaul, Vera Mahler) and Deutscher Allergie- und Asthmabund (DAAB) (Anja Schwalfenberg) were also involved in the consensus process as advisors. 

The guideline was updated during several consensus conferences. Final consensus was reached by all co-authors/delegates on December 6, 2021. This was followed by submission to all societies and involved disciplines for authorization and recommendation for adoption. This final authorization was formally completed by June 30, 2022. 

The guideline is aimed at all physicians with specialization in “allergology” and physicians who treat and/or care for allergic patients who may receive AIT. The guideline applies to all patients with allergic rhinoconjunctivitis (ARC) with/without allergic asthma and allergic sensitization to inhalant allergens. For further information regarding the indication, contraindications, control measures, and duration of therapy in the case of Hymenoptera venom AIT, the AWMF guideline on the “diagnosis and therapy of bee and wasp venom allergy” should be referred to. 

The guideline will be scrutinized by the authors for validity 5 years after publication, with the guideline coordinators being responsible for this procedure. Details can be found in the separate guideline report. 

The guideline is published and disseminated by the allergological societies in their associated publications organs and in the AWMF guideline collection. The guideline is also recommended for adoption by the other societies and disciplines involved, and will be made available for reprinting to interested specialist journals with a focus on allergic diseases. 

## Chapter 2. Immunological mechanisms of AIT 

**Figure 1.**
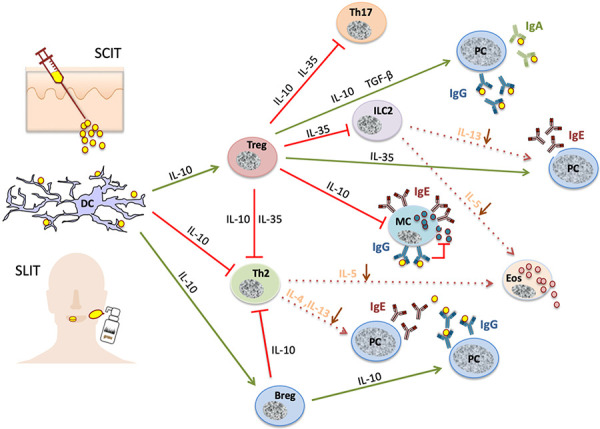
Immunological mechanisms of AIT. On the one hand, AIT leads to activation (green lines) of regulatory lymphocytes and IgG and IgA-secreting plasma cells (PCs), and on the other hand to inhibition (red lines) of different cell populations, which specifically results in suppression of type 2 inflammatory signals such as interleukin (IL-)4, IL-5, and IL-13 (dashed brown lines). Repetitive allergen administration, e.g., subcutaneously (SCIT) or sublingually (SLIT), activates dendritic cells (DCs), which stimulate regulatory T (Tregs) and B cells (Bregs) and inhibit Th2 lymphocytes as well as, consecutively, IgE production and eosinophilic granulocytes. An important key cytokine is IL-10, which additionally promotes the synthesis of allergen-blocking IgG and IgA and suppresses innate lymphoid cells (ILC2), Th17 lymphocytes, and mast cells (MCs). Further important immune-regulatory cytokines are IL-35 and TGF-β. In addition, allergen-fixing IgG antibodies inhibit the secretion of histamine, leukotrienes, and other allergic mediators via binding of inhibitory receptors on MCs. ^©^Authors of the guideline.

AIT induces differential immunomodulation that involves multiple phases and affects both the innate and adaptive immune system (Figure 1). Initially, there is a temporarily reduced reactivity of IgE-carrying effector cells to the allergen stimuli. Subsequently, cellular and humoral immune modifications take place as a sign of a stabilizing and persistent allergen tolerance. 

The early-phase immune mechanisms have not yet been extensively investigated. It is assumed that tissue mast cells develop tachyphylaxis or anergy by a negative feedback mechanism, the main indications for this come from research on basophilic granulocytes (the equivalent cells in peripheral blood). For example, in the context of a rapid, repetitive allergen dose increase (e.g., in ultra-rush or cluster AIT), an effector cell may experience “exhaustion” due to the repeated release of high concentrations of inflammatory mediators (e.g., leukotrienes, histamine). An autocrine suppression may then occur through the binding of histamine to histamine receptor 2, or the inhibition of the effector cells by cytokines such as IFN-γ or IL-10, mechanisms which are currently discussed [2, 3, 4]. 

In the following months, immunological tolerance develops, with the first signs appearing within 1 to several weeks – presumably depending on the induction scheme (either early tolerance induction when the maintenance dose is reached quickly, as in ultra-rush or rush AIT with Hymenoptera venoms, or later through a gradual dose increase as performed with aeroallergens) [3, 5, 6, 7, 8, 9]. Tolerogenic dendritic cells (DCs) are formed, which, after uptake and processing of the applied allergen, transport it to the regional lymph nodes where regulatory T cells (Treg) are stimulated. Interleukin (IL)-10, which is synthesized primarily by T cells but also by B cells and DCs, plays a crucial role here [6]. Among other activities, IL-10 inhibits mast cells, increases the synthesis of allergen-blocking IgG4 antibodies (see below), and suppresses allergic T effector cells, which include not only Th2 but probably also Th17 lymphocytes [10]. Further examples of important immunoregulatory cytokines include TGF-β, which can promote the production of allergen-specific IgA antibodies [3, 11], and IL-35, which can inhibit innate lymphoid cells (ILC2) corresponding to Th2 cells and IgE antibody production [12]. Later in the course of AIT, Tregs decrease and there is an increasing loss of allergen-specific Th2 cells, which leads to the conversion of the Th2 cell-based allergic state to an allergen-tolerant state [8, 9, 12, 13]. This is also reflected by a normalization of the cytokine milieu of the allergic effector organs. Anergy, selective deletion, or apoptosis of Th2 cells have been discussed as underlying mechanisms, among others [13]. 

Furthermore, AIT causes various humoral and B-cell changes. Initially, there is a short-term rise in IgE antibodies and the synthesis of allergen-blocking IgG (especially of the IgG4 subtype) and mucosal IgA antibodies [3, 8]. These immunoglobulins prevent IgE-mediated fixation and presentation of the allergen to T helper cells and thus further activation of Th2 lymphocytes. The allergen binding to IgE on mast cells and basophils is blocked, preventing their stimulation. A direct inhibition of allergic effector cells via the fixation of IgG-allergen complexes to inhibitory IgG receptors has also been discussed [7]. Continued allergen application then leads to a constant increase in allergen-blocking activity in the serum of treated allergic patients and to increasing affinity maturation of these antibodies [14]. These changes occur in a similar way when using native allergen extracts, allergoids, or epitope-specific allergen peptides [3, 8, 15, 16, 17]. B lymphocytes play an additional role in AIT also through the secretion of immunoregulatory cytokines (IL-10, IL-35, and TGF-β) [12, 18, 19].

**Conclusion 1:** The main immune modifications of AIT are i) the temporary induction of regulatory immune cells (DCregs, Tregs, Bregs), ii) the reduction of allergen-specific innate immunity and T helper cell activity, and iii) the formation of allergen-blocking IgG and IgA antibodies. Finally, a ‘T-cell-normalized’ endotype emerges from the primarily Th2-dominated endotype as an immunological prerequisite for clinical allergen tolerance.

## Chapter 3. Allergen extracts, their evaluation and marketing authorization 

### 3.1. Production and composition of allergen extracts 

Allergen extracts differ in composition and allergen activity due to different, manufacturer-specific processing. Even with the same allergen sources, they are therefore not directly comparable. For the allergens that are subject to the German Therapy Allergen Ordinance (TAO, “Therapieallergene-Verordnung”), only standardized extracts are marketable [20]. Total allergenic activity is determined using in vitro methods [21]. The determination of single allergens (usually major allergens) using standardized, validated methods is a long-cherished goal [22]. 

Two recombinant major allergens, rBet v 1 from birch pollen (Betula verrucosa; http://crs.edqm.eu/db/4DCGI/View=Y0001565) and rPhl p 5a from timothy grass pollen (Phleum pratense; http://crs.edqm.eu /db/4DCGI/View=Y0001566) were accepted as reference standards by the European Pharmacopoeia Commission in 2012. These reference standards form the basis of validated standards for determining the Bet v 1 or Phl p 5a content in allergen preparations [23]. 

Immunoassays (ELISA systems) with successfully validated standards and associated antibody pairs are already available internationally (https://inbio.com/elisa-2.0/elisa-2.0-kits-pollen). They are based on ELISA-systems which have been tested in multicenter round robin tests. So far, their use has not been mandatory. The reference allergens Bet v 1 and Phl p 5a have already been included in the European Pharmacopoeia. After the successful inclusion of the ELISA methods for 1) Bet v 1 and 2) Phl p 5a as general chapters in the European Pharmacopoeia and subsequent modification of the Allergen Monograph, the content of these major allergens in single extracts with birch pollen or grass pollen extracts in the future will have to be declared based on the reference ELISAs. 

So far, the allergen concentrations of different preparations could not be directly compared using manufacturer-specific units, especially since the manufacturers often used different standards, antibodies, and measuring methods (“in-house assays”) for determining major allergens. 

**Figure 2.**
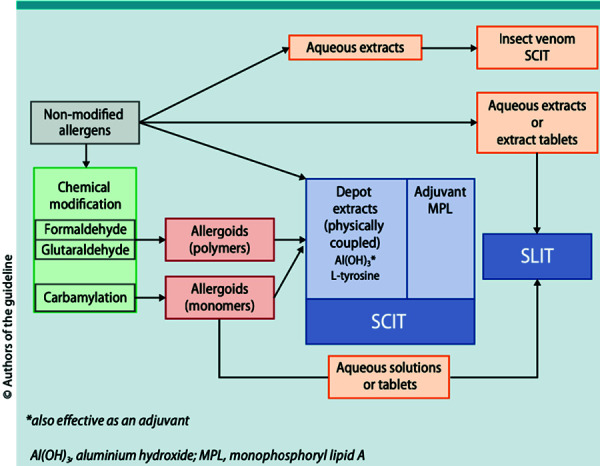
Available allergen extracts for AIT (for an explanation see Section 3.1.).

In general, non-modified (“native”) extracts with an unmodified allergen conformation and chemically modified extracts (allergoids) are available for subcutaneous immunotherapy (SCIT). The latter are based on the concept of having less reactive B-cell epitopes and thus reduced IgE binding, while T-cell epitopes and immunogenic effects should be retained [24]. In addition to aqueous extracts – which are common in the initiation of therapy for insect venom allergies – many semi-depot extracts are used for SCIT in Europe. Here, the allergens or allergoids are physically coupled to a carrier such as aluminium hydroxide [Al(OH)3] or tyrosine [25] (Figure 2). Preparations for sublingual immunotherapy (SLIT) are available with allergens in unmodified or chemically modified conformation as aqueous solutions or tablets (Figure 2). There are preparations that are intended to be stored in the refrigerator as well as products that can be stored at room temperature.

**Conclusion 2:** AIT products (SCIT and SLIT) are not comparable due to their heterogeneous composition. Likewise, the allergen concentrations given by different manufacturers to date are also not comparable due to different methods of measuring the active components. For SCIT, non-modified allergens are used as aqueous or physically coupled (semi-depot) extracts, and chemically modified extracts (allergoids) are used as semi-depot extracts. The allergen extracts and allergoids for SLIT are used as aqueous solutions or tablets. In the future, according to the European Pharmacopoeia, it will be mandatory to indicate the quantity of Bet v 1 in birch pollen extracts and Phl p 5a in timothy grass extracts.

### 3.2. Evaluation criteria of allergen immunotherapy with subcutaneous or sublingual application in clinical studies 

**3.2.1 Primary and secondary outcome measures**

Careful selection of the primary endpoint is essential for demonstrating the efficacy of AIT in randomized controlled clinical trials [26, 27]. The efficacy of AIT is measured using patient-reported outcomes (PROs) such as symptom scores (e.g., individual symptoms; total symptom score (TSS)), medication scores, combined symptom and medication scores (CSMS), quality of life assessments (health-related quality of life (HRQL)), and other methods (e.g., visual analogue scales (VAS), “well days” or “severe days”) [27, 28, 29]. 

The lack of validation of primary and secondary outcome measures and various proposed variants of CSMSs [27] make it very difficult to compare the results of different studies [30]. Since 2008, the European regulatory authority (European Medicines Agency (EMA)) recommends to use a CSMS and accepts (in justified exceptional cases) a positive study result in both individual scores, since the use of medication influences the degree of symptoms, and therefore, in addition to reporting symptoms, also the need for symptomatic medication should be reflected in the score. However, the EMA does not commit itself to a specific CSMS [31, 32]. A task force working group of the European Academy of Allergy and Clinical Immunology (EAACI) has favored a standard for the CSMS as the primary endpoint since 2014 with the aim of harmonization for adults and children in future clinical trials [27, 30]. Recently, an additional responder analysis to assess efficacy has also been proposed [30, 33]. 

In addition, the assessment of laboratory data as potential biomarkers (e.g., IgE, IgG, and IgG4, blocking antibody activities, regulatory T-cell activity, and basophil reactivity) in the placebo and actively treated groups [7, 34, 35] is also reasonable.

**Recommendation 1:** Data on safety and efficacy should be standardized, e.g., by grading according to recommendations of the EAACI or the World Allergy Organization (WAO). In addition, further investigations into possible biomarkers and immunological mechanisms of AIT are useful (*strong consensus, agreement of 100%*).

**3.2.2. Monitoring the effectiveness of AIT under routine conditions**

Few real-world evidence (RWE) studies on the success of AIT under routine conditions are available [36, 37, 38]. There is increasing evidence for the effectiveness and secondary preventive effects of AIT based on prescription and coded data from patients covered by statutory health insurances [39, 40, 41]. An alternative to generate RWE data on the application of AIT outside clinical studies is to use hand-device applications (apps), which are being increasingly utilized by patients and may be helpful in the early stratification of patients for AIT and furthermore in therapy monitoring [42, 43]. For example, based on the data set of the app “MASK-air” (Mobile Airways Sentinel Network [38, 42, 44]), a concept study recently demonstrated that AIT seems to reduce allergic symptoms in routine treatment and to increase the patients’ productivity at work [45]. 

**3.2.3. Allergen exposure**

In order to assess the clinical efficacy of AIT it is further necessary to record the (regional) allergen exposure over time [30, 46]. Definitions proposed by EAACI of the pollen season via pollen concentrations have already been validated for grass and birch pollen in order to be utilized in future clinical trials [47, 48]. For seasonal allergens, EAACI recommends recruiting all study subjects in the same season, measuring outcomes particularly during the peak pollen period, and conducting two identical studies simultaneously in geographically different regions [30, 47]. 

**3.2.4. Data analysis and presentation**

It is essential that all study results are evaluated, presented and published in a suitable form. For this purpose, standards were developed (consolidated standards of reporting trials (CONSORT)) that are intended to guarantee minimal but also transparent information on the studies via standardized checklists (www.consort-statement.org) [49, 50]. This includes the evaluation of the clinical data in an intention-to-treat (ITT) analysis, which takes into account all patients included in a study (including those who drop out very early) to be able to demonstrate the actual effects of AIT under conditions of practice [49, 50, 51]. Per-protocol (PP) analysis, on the other hand, is suitable for estimating the maximum grade of efficacy under optimal standardized conditions. In addition, by analyzing the full analysis set (FAS), data of all patients, i.e., also of those who were included in breach of the inclusion criteria or treated with deviation to the study-protocol, are also recorded for the description of the safety profile of the treatment. 

Since significant placebo responses in clinical AIT studies are the rule rather than the exception and the primary target parameters are PROs [52, 53], it is desirable that in future studies the effects achieved with placebo be explicitly described and in just as much detail as those achieved with the active ingredient [30, 52, 53, 54].

**Conclusion 3:** The clinical efficacy of AIT is measured using patient-reported outcomes (PROs) as primary and secondary endpoints. For clinical phase III studies, the EMA stipulates a combined symptom and medication score (CSMS) for the primary outcome parameter. The CONSORT recommendations specify standards for the evaluation, presentation, and publication of study results. The results of the placebo group are to be described in just as much detail as those of the actively treated group.

### 3.3. Significance of the marketing authorization of allergen preparations 

According to Directive 2001/83/EC, test and therapy allergens are medicinal products in all EU member states and are subject to marketing authorizations. According to Article 5 of this Directive, however, an exemption from these provisions can be made to fulfill special needs. 

The responsible national competent authority for allergen products in Germany is the Paul-Ehrlich-Institut (PEI) based in Langen near Frankfurt/Main. In Austria, marketing authorization is granted by the “Federal Office for Safety in Healthcare” (Bundesamt für Sicherheit im Gesundheitswesen (BASG)), the operative tasks of the BASG were discharged by the “Austrian Medicines and Medical Device Agency” (Medizinmarktaufsicht), a business division of the “Austrian Agency for Health and Food safety” (Österreichische Agentur für Gesundheit und Ernährungssicherheit) (AGES MEA). In Switzerland, the marketing authorization of allergens is supervised by Swissmedic (Schweizerisches Heilmittelinstitut). 

In Germany, the scope of Directive 2001/83/EC is fully implemented in the German Medicinal Product Act (Arzneimittelgesetz (AMG)) [55]. According to this, test and therapy allergens are finished medicinal products and may only be marketed in Germany if they have been obtained a marketing authorization by the national competent authority. In accordance with Article 5 of the EU Directive, there are exemptions that exempt individual formulations (“named-patient products” (NPPs)) for therapy allergens from marketing authorization requirements, even though they are finished medicinal products (Table 1). Approved AIT products and NPPs require a prescription and are marketable. 

Until 2008, therapy allergens from all allergen sources could be marketed in Germany as NPPs without marketing authorization. In 2008, the German TAO came into force with the aim that for therapy allergen products with active ingredients from the most common allergen sources in Germany (Table 2), the quality, efficacy, and safety must be proven without exception in a marketing authorization procedure and these AIT products may no longer be marketed as NPPs without marketing authorization [20]. 

For products that contained the respective allergen sources and that were on the market as NPPs at the time when the TAO came into force in Germany, an application for marketing authorization had to be submitted in order to remain marketable. The marketability of corresponding products for which no marketing authorization was sought ended after a transitional period in November 2011; since then, they have not been any longer on the market in Germany [56]. The first national marketing authorizations were granted in 2018 for two preparations that were assessed and further developed under the development program of the TAO (Table 3). Applications for marketing authorization for further 49 therapy allergens are still pending under the legal transitional provisions (according to the list of “Marketable Therapeutic Allergens according to the TAO” of the PEI; as of April 12, 2022; https://www.pei.de/DE/arzneimittel/allergene/therapie-verkehrsfaehig/verkehrsfaehig-node.html). 

The pre-prepared bulk from which these therapeutic allergens (Table 2) are manufactured are subject to federal batch testing (pre-prepared bulk batch testing), whereas batch testing is carried out on the end-product in authorized preparations where possible. Meanwhile, an official review of the manufacturing process, clinical efficacy, and safety takes only place during the authorization process. Until a decision on the application for marketing authorization under the TAO is made, these products are equivalent to the approved preparations in terms of their ability to be prescribed and marketed. However, if there is a lack of proof for efficacy in the clinical documentation and/or evidence for impaired product safety, further batch release will be denied until the marketing authorization is ultimately refused by the PEI. All other therapeutic allergens produced as NPPs that do not contain any allergens listed in the appendix of the TAO (examples in Table 4) are still exempt from the requirement of marketing authorization and are therefore neither subject to official quality, efficacy, and tolerability controls, nor to federal batch testing. According to the German Medicinal Products Act, a manufacturing authorization is required that ensures compliance with the Good Manufacturing Practice (GMP) criteria. 

Authorized preparations (http://www.pei.de) can be distinguished from NPPs by the marketing authorization number on the outer packaging and in the summary of the product characteristics (SmPC). 

Marketing authorization can only be granted in case of a positive benefit-risk ratio. Among other things, information on the manufacturing of the medicinal product and quality control, the results of all preclinical examinations and clinical trials, and other medical tests are to be submitted to the competent authority together with the authorization documents. For marketing authorization, quality, efficacy, and safety of the medicinal products must be demonstrated according to the current state of the art. The level of knowledge evolves over time, which generally results in increased requirements. This has led to a high quality of data collected in clinical trials and thus to a higher grade of evidence for the efficacy and safety of AIT products authorized on the basis of such studies. The current state of the art includes, for example, GMP, good clinical practice (GCP), the European Pharmacopoeia (Pharmacopoea Europaea), and the corresponding guidelines of the EMA on the manufacturing and quality of allergen products (https://www.ema.europa.eu/en/documents/scientific-guideline/guideline-allergen-products-production-quality-issues_en.pdf) and on the clinical development of AIT products (http://www.ema.europa.eu/docs/en_GB/document_library/Scienti %20c_guideline/2009/09/WC500003605.pdf) [21, 31, 57]: After corresponding dose-finding studies (phase II) and confirmatory clinical trials (phase III), AIT preparations are currently only authorized for those indications and patient groups for which efficacy and safety compared to placebo have been proven. For ethical reasons, no placebo control is required for the authorization of Hymenoptera venom AIT preparations; the comparator in this case is usually an established reference product. 

A current overview of clinical trials on AIT approved in the European Union can be found in the EU Clinical Trials Register at www.clinicaltrialsregister.eu. The manufacturer can present results on the efficacy obtained in corresponding studies (the quality of which can vary significantly from 1990 to the present day due to different requirements) in section 5.1 of the SmPC. In the case of authorized preparations, this information in the SmPC has also been reviewed by the authorities. With current marketing authorizations, this option is used by the manufacturers and offers the physician a good opportunity to obtain information on this preparation. 

Since authorized finished medicinal products cannot cover the full range of allergen extracts needed for AIT (particularly for less common allergen sources), NPPs retain their justification for low-prevalence allergies for which a sufficient number of patients for clinical trials cannot be reached (Table 4) [58].

**Conclusion 4:** Products containing frequent allergen sources (pollen from sweet grasses (except maize), birch, alder, hazel; house dust mites; bee and wasp venom) must obtain a marketing authorization in Germany according to the Therapy Allergen Ordinance. During the authorization process, quality, efficacy, and safety of these products are assessed. Authorized or otherwise marketable allergen products demonstrating a positive risk-benefit ratio according to EMA guidelines should preferably be applied. Named-patient products are used to prescribe rare allergen sources for AIT. They cannot be mixed with the allergens listed in the Therapy Allergen Ordinance. Country-specific regulations apply to Austria and Switzerland.

**Recommendation 2:** Authorized or otherwise marketable allergen preparations demonstrating a positive risk-benefit ratio according to EMA guidelines should preferably be applied (*consensus, agreement of 94%). *

### 3.4. Socio-economic aspects of AIT 

Allergic diseases, including ARC, allergic asthma, and allergic skin diseases, have a significant impact on the health of the individual patient, but also on healthcare costs and the economy as a whole [59, 60, 61, 62]. Not only the costs directly related to the disease, but also the indirect and intangible costs put a burden on those affected and the healthcare systems [60, 63]. The direct disease costs for ARC already totaled several hundred million euros as early as the 1990s [61]. The high overall costs of ARC do not so much result from high *per capita* costs but rather from the high prevalence of this disease. The intangible costs of allergic diseases are essentially determined by the high grade of impairment to quality of life. 

The guideline-based treatment of allergic diseases serves to create a high level of patient benefit by reducing the burden and progression of the disease and by improving the quality of life. Treatment options include symptomatic therapy, allergen avoidance, and in many cases also AIT as disease-modifying form of treatment. From an economic point of view, the cost-effectiveness of AIT is based on its clinical efficacy and the patient benefit at reasonable costs [64]. According to current studies, the individual course of the disease can be favorably improved by both the curative and preventive properties of AIT (*disease-modifying effect*). Patients with allergic rhinitis have a 3.5-fold increased relative risk of developing bronchial asthma within less than 10 years [65]. There are several studies in the literature with supporting evidence for AIT as a disease-modifier by reducing disease progression (to allergic bronchial asthma) ([66, 67], see also Chapter 4). 

The health economic evaluation of therapeutics is based on analysis of a) cost-benefit, b) cost-effectiveness, or c) cost-utility. These analyzes allow a comparison of different therapy methods and single products and an evaluation of the advantages and disadvantages from an economic point of view. All forms of analysis are reported in the current literature on AIT [60]. In a) costs versus costs are analyzed, in b) the costs are compared with natural clinical outcomes, such as a clinical score, and in c) costs and patient-reported endpoints such as quality of life are weighed against each other. 

The results of such analyses are used in the evaluation of preparations and play an important role in the decision by the healthcare systems to support reimbursement. In many countries, but not in Germany, the quality of life gained per year after AIT is determined using the standardized “quality-adjusted life year” (QALY) and used for incremental cost-benefit analyzes [68]. Each year of life in perfect health is expressed with a QALY of 1, diminishing according to disease burden to a QALY of 0.0 for death. By dividing the disease course, the difference in costs for different procedures or timepoints in treatment (in this case AIT) by the relevant QALY, one obtains the incremental cost-effectiveness ratio (ICER). 

Recent studies have shown that the ICER for AIT, irrespective of its routes of application, falls within the range for the treatment of chronic diseases [69, 70]. Another cost-effectiveness analysis from Germany underlines the cost-saving potential of AIT [71]. The improvement in the treatment rate in the indication groups is of additional economic importance [72]. 

Meta-analyses also found no relevant difference between the sublingual and subcutaneous routes of application [73]. However, the nature of the costs impacts these analysis [74]. There are also a large number of international studies demonstrating the economic efficiency of AIT [75, 76, 77]. Based on the cumulative ICER per year, a long-term analysis revealed that the significant investment at the beginning of treatment proves to be cost-neutral after 7 years on average [78]. It should be emphasized that these effects are strongly dependent on treatment compliance. Generally speaking, the prices of individual products valid at the time (according to the official drug price list (LAUER-TAXE^®^) and at dosage according to the manufacturer’s recommendations) for a treatment period of 3 years ought to be used to compare the costs of SCIT and SLIT.

**Conclusion 5:** Allergic rhinoconjunctivitis (ARC) and allergic bronchial asthma cause considerable direct, indirect, and intangible costs for society as a whole. AIT is significantly more cost-effective over the long term when indicated and used in accordance with guidelines compared to pharmacotherapy alone provided that adherence to therapy is good. The choice of the AIT product has to be decided on an individual basis, whereby clinical benefits are given priority over costs according to German social law.

## Chapter 4. Efficacy of AIT 

### 4.1. Systematic reviews and meta-analyses evaluating the efficacy of AIT 

A systematic review summarizes the medical literature using defined and reproducible methods for literature search and carries out a critical evaluation. In contrast, meta-analysis is a mathematical-statistical synthesis of many studies aiming to estimate effect size through the summation of different analyses. In addition to the effect sizes only, meta-analyses report the distribution of values to the mean. The term *meta-analysis* ought to be only used when established statistical methods such as appropriate calculation of effect size, weighting, and analysis of heterogeneity as well as statistical models taking into account the different hierarchical structure of meta-analytic data have been followed [79, 80, 81]. With the *Preferred Reporting Items for Systematic Reviews and Meta-Analyses (PRISMA)* statements, consented standards for the presentation (good reporting practice) and language of systematic reviews and meta-analyses in evidence-based medicine have been established [82, 83]. 

In evidence-based medicine, there is a consensus that meta-analyses are at the top of the pyramid in the evidence hierarchy [84]. However, meta-analyses have also been critized, particularly when they include studies of low quality or high heterogeneity and neglect possible publication-biases (studies reporting no or no significant effect are rarely published) [84]. It has recently been reported that many authors of systematic reviews and meta-analyses have reported not to thoroughly follow all critical methodological steps [85]. Despite their usefulness, meta-analyses are limited regarding scientific synthesis and decision-making [79]. Although meta-analyses can shed light on areas in which the evidence is insufficient, they cannot compensate for this deficiency *(“They are statistical and scientific techniques, not magical ones.”)* [79]. One way to reduce the heterogeneity of the study results and at the same time to make statements relevant to daily routine practice is to strictly select the studies to be included in the analysis according to predefined criteria [1, 27, 86]. 

For example, it would be possible to include only AIT studies with at least 100 subjects per arm or those which use standardized instruments for reporting results according to CONSORT [87, 88, 89] or which apply for example, a CSMS [27, 86]. It should be noted that constraints always contain a potential for bias. 

Meta-analyses on AIT have repeatedly been carried out, and in the more recent meta-analyses more studies with a large number of high quality cases could be included. Overviews of the published meta-analyses up to and including 2009 can be found in [90] and [91]. The most recently published indication-related meta-analyses and the number of included studies as well as the year of publication can be found in Table 5. These results of systematic reviews and meta-analyses are taken into account in national and international guidelines as well as in clinical pathways [30, 33, 38, 92, 93]. 

Nevertheless, meta-analyses are not able to answer many practical questions about AIT in the daily patient management [94, 95]. Other parameters that are important for assessing the evidence of AIT in clinical studies, such as the drop-out rate [96], adherence [97], or the effects achieved with placebo [30, 52, 53, 54] have also rarely or not been considered in meta-analyses. 

In summary, the meta-analyses confirm the well-documented efficacy of AIT in allergic rhinitis/rhinoconjunctivitis, allergic asthma, and insect venom allergy. However, due to the heterogeneity of the studies reported in all analyses, the authors emphasize that a generic recommendation in the sense of a class effect is not possible in AIT, but that specific evidence of efficacy and tolerability is required for each single AIT preparation separately.

**Conclusion 6:** Systematic reviews and meta-analyses demonstrate the efficacy of SCIT and SLIT for certain indications, allergens, and age groups. The data from the controlled studies differ significantly in terms of their scope, quality, preparations, and dosing regimens and require a product-specific evaluation. A broad transfer of the efficacy of certain preparations to all preparations administered in the same way is not endorsed.

### 4.2. Product-specific evaluation of the AIT products marketed in Germany and/or Switzerland and/or Austria (homologous groups of grass, tree pollen (Betulaceae) and house dust mite allergens) 

Due to the above-described high level of heterogeneity in the clinical documentation of AIT products a product-specific evaluation of the AIT products on the market in Germany, Switzerland, and Austria is recommended. Based on the present update of the S2k guideline, the tabular presentation of individual products on the homepage of the DGAKI has been updated in a modified form (https://dgaki.de/leitlinien/s2k-leitlinie-ait/). This tabular presentation will be updated every 6 months. The listing is based exclusively on the information provided by the German and European authorities PEI and EMA, the study register clinicaltrialsregister.eu as well as the referenced scientific full-publications of the respective therapy allergens. 

This list includes the following features for the AIT products (separated according to the three homologous groups, in alphabetical order): i) the year of marketing authorization if applicable, as well as further details of the authorization (national authorization procedure before the TAO, national authorization procedure under the TAO, European authorization procedure by the EMA, authorization for the treatment of children, authorization for the treatment of adolescents), ii) overview of studies for products with currently marketed dosages (phase II and III studies) and information on publications, if available, and iii) overview of studies for products following the TAO regulation procedure (phase II and III studies) and information on the publications, if available.

**Conclusion 7:** A product-specific evaluation of the individual AIT preparations according to clearly defined criteria is recommended. On the DGAKI website (https://dgaki.de/leitlinien/s2k-leitlinie-ait/) a tabular overview with AIT product-specific information is given, which includes the homologous groups of grass, tree pollen (Betulaceae), and house dust mite allergen preparations distributed in Germany and/or Austria and/or Switzerland.

### 4.3. Grass pollen allergy 

Respiratory allergies (allergic rhinitis/rhinoconjunctivitis, allergic asthma) due to grass pollen allergy are among the most common forms of allergy in our climate. The grass family (Poaceae) exist all over the world. The subfamily of the Pooidae (“temperate” grasses) includes the grasses that are predominantly native to our latitudes and whose pollen allergens are highly cross-reactive. Subtropical grasses (Panicoideae/Chloridoidae) can be found particularly in warmer climate zones, the pollen of which shows only partial cross-reactivity with the pollen of the grasses native in our latitude [107]. However, tropical grasses also occur in Europe [108]. When counting pollen, it is not possible to distinguish between the pollen of individual species. Data on the pollen load of subtropical grasses growing here are not available for Germany. 

Because of the extremely long flowering phase of the Poaceae, from around April until September, grass pollen allergy is a serious burden for many patients and significantly impacts their quality of life. AIT represents the only causal form of treatment and complements symptomatic therapy. For AIT, mixtures of pollen from many or a few grass species belonging to the Pooidae subfamily are on the market, with no significant difference in allergenic activity [109] even if the composition of the grass pollen extracts is different and standardization of the extracts is performed on the basis of the major allergen compounds of group 1 or group 5 [9]. 

Allergic rhinitis is a major risk factor for the development of allergic asthma [65]. Here, AIT with grass pollen allergen represents a possible preventive treatment in addition to its complementary effect with anti-symptomatic medication. The following is an overview of the current evidence on the efficacy and safety of AIT for grass pollen allergy in patients with seasonal ARC and allergic asthma. 

However, there remains a high degree of heterogeneity in the clinical documentation and evidence for the various products used in AIT with regard to their efficacy and safety. It is therefore recommended regarding grass pollen extracts (and the same holds true for all other allergen extracts which will be reported in subsequent chapters) to evaluate the different AIT products on the basis of the clinical development programs and their efficacy and safety documented in clinical studies (product-specific evaluation). 

**4.3.1. Efficacy of AIT in ARC and grass pollen allergy**

*SCIT*

There are many clinical studies in the literature with evidence of efficacy of SCIT in the treatment of adult patients with grass-pollen related ARC (including [17, 98, 110, 111, 112, 113, 114, 115, 116, 117, 118]). 

Not all grass pollen extracts available or with market-authorization have been tested according to the WAO and EMA efficacy criteria, and there are no specific pediatric studies for most of the preparations. An open-label, non-controlled, multicenter study including 284 children found no significant differences in the efficacy of 6 different SCIT preparations approved in Germany [119]. 

In children and adolescents with ARC, a SCIT preparation with birch or grass allergens or a birch-grass mixture, in addition to reducing the symptoms of ARC, was able to achieve a reduction in the risk of developing allergic asthma in a prospective, open-label study (“*preventive allergy treatment* (PAT) study” [66]). This effect was still observed 7 years after the end of SCIT compared to the control group that only received symptomatic treatment with pharmacotherapy [120]. Using prescription and coded data, an RWE study demonstrated a statistically significant protective effect of AIT on the incidence of bronchial asthma in patients with allergic rhinitis and an allergy to seasonal allergens [121]. Another RWE study showed a statistically significant reduction in allergic rhinitis medication of 64.8% after grass pollen SCIT with a similar effect of 60.7% in children [39].

**Conclusion 8:** The efficacy of SCIT in ARC in grass pollen allergy has been demonstrated very well by numerous studies in adult patients; in children and adolescents, this has been proven by few studies. An uncontrolled trial and an RWE study showed asthma-preventive effects in children and adolescents. In general, there are product-specific differences in the documentation of clinical efficacy which underlines the importance of a product-specific evaluation. This also applies to all of the following allergen groups.

*SLIT*

The efficacy of SLIT with grass pollen extracts in ARC with or without concomitant asthma has been documented in several large studies conducted in Europe [122, 123] and the USA [124, 125]. There is a high level of evidence for the clinical efficacy (in terms of the number and methods of the studies) for the sublingual tablets that have already received marketing authorization [122, 123, 126]. 

Studies investigating grass tablets over one season in grass pollen-allergic children from the age of 5 have demonstrated comparable effect-size to adult studies [127, 128, 129]. Both preparations have therefore also been approved for children from the age of 5 years. 

For both grass tablets currently available on the market, a “*carry-over*” effect has been demonstrated in adults: clinical efficacy was maintained 1 [130, 131] or 2 [132] years after the end of a 3-year treatment period. For one grass tablet, a carry-over effect was shown 2 years after the end of 3-year continuous treatment in a double-blind, placebo-controlled study for children aged 5 years and older [67]. Large double-blind placebo-controlled (DBPC) trials have also shown liquid grass SLIT preparations to be clinically effective in both children and adults [133, 134, 135, 136]. Study results for other liquid grass SLIT preparations are contradictory or these preparations were not examined at all in DBPC studies. 

Also for SLIT, a preventive effect with regard to the involvement of the lower respiratory tract (asthma development) has so far mostly been demonstrated in open-label studies [137, 138, 139]. The only prospective, controlled study on asthma prevention so far was carried out in over 800 children and adolescents with grass allergy and only rhinoconjunctival symptoms at the study initiation. 3-year AIT with a grass tablet (SLIT) led to a significant reduction in asthma symptoms and asthma medication compared to placebo treatment from the 2^nd^ year of treatment until 2 years after treatment cessation, i.e., 5 years after the start of the study (“*Grass Sublingual Immunotherapy Tablet Asthma Prevention (GAP)*”) [67]. However, no significant difference was found in the first onset of asthma, pre-defined as documented reversible pulmonary obstruction (primary endpoint). 

An RWE study showed a statistically significant impact of SLIT in terms of reduced prescription of medication for allergic rhinitis in both adults and children/adolescents between the ages of 5 and 18 [40]. Another RWE study showed a statistically significant reduction in medication for allergic rhinitis of 53.6% after grass pollen SLIT, with a similar effect in children [39]. In addition, an RWE study for Germany using two different grass pollen tablets showed a 50% reduction in the number of prescriptions for medication for allergic rhinitis following a SLIT treatment course of at least 2 years [140].

**Conclusion 9:** The efficacy and safety of SLIT in ARC caused by grass pollen allergy in adults and children is very well documented. However, product-specific differences exist. A controlled study has indicated asthma-preventive effects in children and adolescents.

**4.3.2. Efficacy of AIT in allergic asthma and grass pollen allergy**

*SCIT*

In contrast to the use of SCIT in ARC, the indication for SCIT in allergic bronchial asthma is usually more restrained [86, 141, 142, 143]. SCIT is not a substitute for adequate anti-asthmatic therapy. Based on numerous studies, SCIT can be recommended in cases of mild to moderate asthma (classification according to the Global Initiative for Asthma (GINA) 2020 [143], the German Respiratory Society (DGP) and German Respiratory League [141]), if the allergic component of the asthmatic symptoms is well documented, and confirmed by allergic sensitization with clear clinical symptoms after exposure to the respective allergen. This also corresponds to the recommendations of the German “Nationale Versorgungsleitlinie Asthma” [142] based on meta-analysis data of the Cochrane Library [144]. The latter included 88 randomized, controlled, but methodologically heterogeneous SCIT studies with a total of 3,459 patients with allergic asthma to house dust mite allergens (42 studies), pollen allergens (27 studies), animal allergens (10 studies), and other allergens. The analysis of all evaluated studies showed a significant reduction in the symptom score and the consumption of medication. Furthermore, there was a slight but significant reduction in non-specific bronchial hyperreactivity. The significant reduction in allergen-specific bronchial hyperreactivity to house dust mite allergens as well as to pollen and animal allergens in patients with SCIT compared to the control groups can be interpreted as a lower relative risk of asthma exacerbation when exposed to the relevant allergen. In 20 of the included studies, lung function parameters were analyzed: there was a trend towards an improvement in lung function, but this did not reach significance [144]. However, since patients with intermittent or mild persistent asthma usually do not show any significant impairments in lung function parameters, this clinical endpoint is not suitable for assessing the efficacy of SCIT. Unfortunately, no separate analysis was performed for children in this Cochrane study. 

The relatively small group of patients with insufficiently controlled asthma represents a risk group for systemic side effects resulting in a careful indication and initiation of AIT [1, 92]. The most comprehensive systematic review and meta-analysis on AIT in bronchial asthma, published in 2017, included 89 DBPC trials [100]. In 9 SCIT studies that could be included in this meta-analysis, there was a strong effect in improving the symptom score both in relation to all allergens (grass pollen, tree pollen, animals, mold) (standard mean difference (SMD) –1.64, 95% CI –2.51 to –0.78) and in the grass pollen subgroup (SMD –1.18, 95% CI –2.17 to –0.20; 4 trials). This effect was also found in children and adolescents under the age of 18. A strong effect was also found from 7 studies in the improvement of the medication score related to all allergens (SMD –1.65, 95% CI –2.52 to –0.79), an implied (but unconfirmed) effect was demonstrated in the subgroup of grass pollen (SMD –0.06, 95% CI –0.41 to 0.28), but only 2 studies could be evaluated here. For the secondary endpoints, there was a strong effect regarding improvement of allergen-specific bronchial hyperreactivity for the overall group of all allergens (SMD 0.93, 95% CI 0.08 to 1.79; 3 studies), the latter also supported by 8 high-quality RCTs and a significant improvement in disease-specific quality of life (SMD –0.83, 95% CI –1.19 to –0.47; 3 studies). In a DBPC study with 35 children and adolescents aged between 3 and 16 years with seasonal grass pollen-associated asthma that was included in this meta-analysis [100], it was shown that SCIT with an unmodified (native) allergen extract can reduce the asthma symptom-medication score significantly [145]. 

In an open-label, uncontrolled, multicenter study including 284 children with ARC with and without bronchial asthma, no significant differences in the efficacy of 6 different SCIT preparations approved in Germany were observed in asthmatic children [119]. An RWE study showed a statistically significant reduction in the prescription of asthma medication by 14.0% after 3 years of treatment, and by 27.4% in children [39].

**Conclusion 10:** The efficacy of SCIT with grass pollen extracts in seasonal allergic asthma caused by grass pollen allergy has been proven well in adult patients and has been proven in children only in a few studies.

*SLIT*

Compared to ARC, there are only a limited number of studies on the efficacy of SLIT in patients with bronchial allergic asthma. The available data mostly come from subgroup analyses of studies on the efficacy of AIT in ARC that also included patients with additional, concomitant bronchial asthma. A new and important approach are studies on the efficacy of AIT preparations in children and adults with bronchial asthma that analyze as the primary endpoint the maintenance of a good asthma control during a stepwise reduction in the daily dose of inhaled corticosteroids [146]. 

In a systematic review and meta-analysis of the EAACI on AIT in allergic asthma published in 2017, the subgroup analysis of patients using SLIT in short-term studies revealed only a questionable benefit in terms of reduction in symptom scores (SMD –0.35; CI –0.75 to 0.05) and medication scores (SMD –0.29; CI –0.82 to 0.24) [100]. However, only 6 double-blind, placebo-controlled SLIT studies were included, most of which were older (published between 1999 and 2009). Only two of these studies looked at grass pollen SLIT in asthma, one in adults showing no significant effect [147] and one in children and adolescents with a positive effect [148]. Overall, the included studies with aqueous cat, mite, and grass pollen extracts had a very high level of heterogeneity. Since most of the analyzed products no longer meet the current standards, the result of the meta-analysis can only be used to a limited extent for deriving current recommendations. Further study results on the effect of modern SLIT preparations on allergic bronchial asthma are expected to be available within the next few years. 

The aim of an AIT intervention should be to reduce the medication necessary to maintain good asthma control and to mitigate the risk of asthma exacerbations. In this regard, an RWE analysis found a statistically significantly reduced prescription of asthma medication in patients with existing bronchial asthma. Furthermore, the data suggested a reduced risk of developing asthma during AIT based on a statistically significantly reduced level of first prescription of asthma medication [40]. Another RWE study also showed a statistically significant reduction in the prescription of asthma medication after 3 years of therapy by 10.6% in all age-groups, and by 21.0% in children [39]. In an RWE study with two different grass pollen tablets, the relative risk of an initial prescription for asthma therapy following SLIT was significantly lower at 62.5% in the SLIT group, as was the number of prescriptions for asthma medication in existing asthma [140]. 

A more recent RWE study, based on the asthma medication prescribed according to GINA severity, was able to demonstrate a significant reduction in asthma progression through AIT, which, however, was not specified further. This effect was greater in adolescents and young adults than in the total population [149].

**Conclusion 11:** There are only very few representative studies on the efficacy of SLIT in adults with seasonal bronchial asthma induced by grass pollen allergy, and few representative studies in the age groups of children and adolescents. Based on the current data, there is only limited evidence to recommend SLIT in allergic asthma due to grass pollen.

**Recommendation 3:** In seasonal ARC induced by a grass pollen allergy, AIT, if indicated, should be carried out in adults and children/adolescents only with products with documented efficacy. In the case of well or partially controlled seasonal bronchial asthma due to grass pollen allergy, AIT ought to be performed in adults and children/adolescents when indicated (*strong consensus, agreement of 100%*).

### 4.4. Tree pollen allergens (Betulaceae) 

Birch/hazel/alder/oak/beech pollen all are from the beech family (Fagales) and show a high grade of cross-reactivity, with birch representing the most relevant source of allergens. Evidence exists that AIT with a birch pollen extract also reduces the symptoms during the hazel and alder pollen season, and an effect on the improvement of oak-pollen related symptoms has also been demonstrated [150]. 

**4.4.1. Efficacy of AIT in ARC and tree pollen (Betulaceae) allergy**

*SCIT*

In a series of DBPC studies on the efficacy of AIT on birch pollen allergies, a reduction in symptoms and/or medication use was shown for some preparations [98, 151, 152, 153, 154, 155, 156]. On the contrary, the safety and efficacy of many other marketed birch pollen extracts have never been demonstrated in DBPC trials. Specific pediatric studies are lacking for all preparations. 

A German RWE study found that 28.6% (statistically significant) fewer symptomatic medications were prescribed during the first 6 years following prescription of birch SCIT [41]. Another RWE trial indicated an even more pronounced, statistically significant effect after 3 years of AIT: in the overall group of patients who received SCIT with tree pollen extracts, the number of prescriptions for symptomatic allergy therapy was reduced by 56% [39]. In children, the number of prescriptions decreased by 42.3% (statistically significant).

**Conclusion 12:** The efficacy of SCIT in ARC caused by tree pollen (Betulaceae-) allergy in adults has been well documented by numerous studies, whereas there is a lack of specific studies in children and adolescents. First real-world analysis data based on health insurance prescription are indicating efficacy in all age groups.

*SLIT*

Tablet and drop preparations for SLIT in patients with birch pollen allergy have been authorized according to modern standards. A first DBPC study with a birch pollen extract presented a significant reduction in symptom and medication scores compared to placebo after 1 year of therapy [157]. Another trial published in 2014 including more than 570 adult patients with birch pollen allergy found a statistically significant superiority of a liquid tree pollen extract over placebo in pre-/co-seasonal SLIT-course over a period of 2 years [158]. In a randomized study, the safety of a rapid dose increase for this preparation was also confirmed in children and adolescents aged 6 – 14 years [159]. 

After dose-finding studies on a sublingual, already marketed, liquid preparation [160], revealed significant differences of the effectiveness for higher allergen concentrations compared to placebo, a new formulation with higher allergen concentrations was further developed and investigated. The subsequent DBPC trial including 406 adults confirmed a statistically significant and clinically relevant reduction in the combined symptom-medication score with a favorable safety profile [161]. Studies on children and adolescents are still lacking for this preparation. 

The safety and efficacy of a newly developed birch pollen tablet were also demonstrated in a pivotal phase III study in 634 subjects aged 12 – 65 years. Of the 634 subjects, 60 were adolescents [162]. In this study, a positive effect was also found regarding the improvement of symptoms during the alder and hazel pollen seasons though treatment was only carried out with a birch pollen extract. 

For many older liquid tree pollen (birch or birch/alder/hazel mixtures) SLIT preparations, either heterogeneous study results are available or they have not yet been investigated in DBPC trials. 

For tree pollen allergy, RWE data also reported a statistically significant decrease in the number of prescribed symptomatic allergy medication by 46.5% in the whole data-set [39]; in children and adolescents the decrease was 36.8% and also reached statistical significance. The same results were mirrored up to 6 years after cessation of AIT (32.9%, statistically significant) [41].

**Conclusion 13:** The safety and efficacy of SLIT in ARC induced by tree pollen allergy are very well documented in adult patients. For children and adolescents with tree pollen allergy, a few studies indicate that the treatment is safe and efficacious.

**4.4.2. Efficacy of AIT in allergic asthma and tree pollen (Betulaceae) allergy**

There are only a few studies available on AIT in allergic asthma and tree pollen allergy. 

*SCIT*

In the 4 current DBPC trials on SCIT preparations, 25 – 40% of the patients with ARC had concomitant (controlled) birch pollen-associated asthma [153, 154, 163, 164]. Results on asthma symptom control were published in only 1 of these 4 studies. In this trial, a slightly improved, but significantly better asthma control test was demonstrated during the birch pollen season in the actively treated study arm compared to placebo. In all 4 studies, an increased frequency of side effects in asthma patients under AIT has not been reported. In particular, there were no asthma attacks or cases of anaphylaxis. A placebo-controlled study with a limited power showed a lower bronchial allergen-specific hyperreactivity after 1 year of treatment [151]. 

In an RWE study, a 32% reduction in the number of asthma medication prescriptions was revealed over 6 years after the SCIT with birch pollen allergens [41]. These effects have also been demonstrated in another RWE study after 3 years of treatment (overall 9.3%, children 14.1%, both statistically significant) [39].

**Conclusion 14:** A DBPC trial indicates that SCIT with tree pollen extracts is effective in adults with seasonal allergic asthma caused by tree pollen (Betulaceae) allergy. The efficacy of SCIT with tree pollen extracts in adults and in children with seasonal allergic asthma has been investigated only to a low extent. However, data from real-world analysis based on health insurance prescription data suggest efficacy in this indication.

*SLIT*

In 3 DBPC trials investigating SLIT preparations, 20 – 40% of included patients were patients with controlled asthma [158, 161, 162]. Results on asthma symptom control are available from 2 of these 3 trials. One of the studies demonstrated a slightly but significantly better asthma control test during birch pollen season. However, this was not confirmed for the entire pollen seasons of alder, hazel, and birch. The second study found no difference in asthma symptom control between the actively treated and the placebo group. In all 3 studies as well as in a pediatric trial with children aged 6 – 14 years, there were no increased side effects in asthmatic patients, in particular no asthma attacks or anaphylaxis. An RWE analysis found reduced asthma medication prescriptions for existing bronchial asthma during the 6-year follow-up after SLIT (41.2%, statistically significant) [41]. In contrast, another RWE could not confirm such an effect [39]. 

In addition, in comparison to the untreated control group a significant improvement in terms of a lower number of first prescriptions of asthma medication the AIT-course was reported. However, during the 6-year follow-up period after cessation of AIT these effects could not further be demonstrated [41]. 

Clinical trials primarily investigating efficacy of SLIT in birch pollen allergic asthma were not found.

**Conclusion 15:** SLIT with tree pollen can be safely applied in patients with controlled asthma. However, the studies on ARC showed divergent effects on asthma symptom control.

**Recommendation 4:** In ARC to tree (Betulaceae) pollen, AIT should be carried out with tree pollen extracts in adults if indicated, using preparations with demonstrated efficacy. AIT with tree pollen may be considered to be performed in children and adolescents. In well or partially controlled seasonal asthma due to Betulaceae allergy, AIT may be considered to be applied in adults and children/adolescents. (consensus, agreement of 89%)

### 4.5. Allergy to other tree pollen (non-Betulaceae) 

Other clinically relevant tree pollen allergens-sources that do not cross-react the birch and beech families causing ARC and asthma in spring and summer, are, for example, ash, cypress, and plane tree pollen. AIT extracts with these allergens are not regulated by the TAO and there are no high-quality studies. 

The availability of therapeutic extracts is often limited. Sometimes homologous allergen sources (e.g., olive pollen for the treatment of ash tree allergy) can be used.

**Conclusion 16:** For other tree pollen not cross-reactive to the birch and beech families, such as ash, cypress, and plane tree pollen, little evidence is available for clinical efficacy, and the availability of therapeutic extracts is limited.

**Recommendation 5:** AIT using tree pollen not cross-reactive to the birch and beech families may be considered to be carried out in individual cases, even though a general recommendation is not possible in the light of current evidence (see Chapter 3.3 keyword “*named-patient products”*). The clinical relevance of an identified sensitization should be evidenced before AIT is initiated. (*strong consensus, agreement of 100%*)

### 4.6. Allergy to house dust mites 

House dust mites (mainly *Dermatophagoides pteronyssinus* and *Dermatophagoides farinae*) are common worldwide. Most studies on AIT against house dust mites were performed with allergen extracts from these two species (“house dust mites”); only a few studies have examined the efficacy of therapy using extracts from other mite species, especially storage mite species. However, there is a lack of dose-finding trials for these extracts. There is a high grade of cross-reactivity between the house dust mite species, but only to a small extent with storage mite allergens [165]. Most patients are sensitized against both house dust mites, but in Central Europe, at least 11% show skin test reactions only to *Dermatophagoides pteronyssinus* and 5% only to *Dermatophagoides farinae* [166]. 

**4.6.1. Efficacy of AIT in ARC and mite allergy**

*SCIT*

Only a few studies have been published on the dose-response relationship for house dust mite extracts used for SCIT, and most of the studies do not meet modern methodological quality criteria [167, 168, 169, 170, 171]. Due to the lack of dose-finding studies for most preparations, it is unclear whether AIT using these extracts is carried out with the optimal dosage (in terms of safety and efficacy). The efficacy of SCIT with various house dust mite extracts has been demonstrated in DBPC studies [169, 172, 173, 174]; however, the clinical documentation for marketed products differs considerably. Results were summarized in a meta-analysis from 2019 [175]. Only one study performed a direct head-to-head comparison of two different house dust mites extracts and found no significant difference regarding their clinical efficacy [176]. Few pediatric studies (including [177]) of SCIT using house dust mite extracts have been published. An RWE study demonstrated a statistically significant reduction in the number of drugs prescribed for treatment of ARC after at least 2 years of treatment with a house dust mite allergoid in both, children and adults [178].

**Conclusion 17: **Data are available on the safety and efficacy of SCIT using house dust mite extracts in ARC for adult patients. The safety and efficacy of this treatment in children has been demonstrated in studies with small numbers of cases, but a lack of double-blind, placebo-controlled SCIT studies following modern methodological standards remains.

*SLIT*

Efficacy of SLIT in ARC: Dose-finding studies of high methodological quality have been published for some SLIT house dust mite extracts. The primary endpoints were the symptom-medication score under real-life conditions [179, 180], standardized allergen challenge in an exposure chamber [181] or the conjunctival challenge [182], as well as immunological changes in the production of IgE-blocking factors [183]. 

On the efficacy of SLIT using house dust mite extracts in ARC, large, adequately powered studies of high methodological quality have been published for some preparations in recent years [180, 184, 185, 186, 187, 188, 189]. Two house dust mite tablets have received marketing authorization for Germany. A meta-analysis has analyzed data taken from several trials [190]. Overall, the effect sizes found for SLIT with house dust mite extracts are smaller than in the studies with pollen extracts (see above). Pediatric studies have also been published [185, 191, 192, 193], however the dosages investigated were partially lower than those for adults [185].

**Conclusion 18: **For some house dust mite SLIT preparations, the efficacy and safety in ARC in adult and adolescent patients have been proven in studies with large numbers of cases. Studies have also shown the efficacy and safety of SLIT in children with ARC and house dust mite allergy.

**4.6.2. Efficacy of AIT in allergic asthma and mite allergy**

*SCIT*

The efficacy and safety of SCIT using house dust mite extracts in adults with house dust mite-related asthma has been demonstrated in several clinical trials; symptom scores [194, 195, 196, 197], use of medication [194, 195, 196, 197, 198], allergen-specific bronchial hyperreactivity [194, 198], and quality-of-life parameters [194, 197] served as clinical endpoints. Clinical effects of SCIT using house dust mite extracts in house dust mite-allergic children have been confirmed in some studies [199, 200], although the studies were not placebo-controlled; the endpoints were allergen-specific bronchial hyperreactivity [199] or reduction in inhaled corticosteroids [200]. An RWE analysis reported a significantly lower number of first-time asthma drug prescriptions after at least 2 years of treatment with a house dust mite allergoid in both children and adults [178]. Similar effects were found in an earlier RWE study [121]. Furthermore, up to 6 years after treatment cessation, the number of asthma medications prescribed for existing bronchial asthma was significantly reduced in children and adults [178]. This effect was particularly pronounced in children.

**Conclusion 19:** For adults, the efficacy and safety of SCIT for allergic asthma caused by house dust mite allergy has been proven in studies. However, the clinical endpoints used in the studies differ significantly and therefore a comparison of the effect sizes as demonstrated in different studies is not possible. The data for children and adolescents are limited, although evidence exists for efficacy and safety of house dust mite SCIT in allergic asthma in this age group.

*SLIT*

The safety [201] and efficacy of SLIT in at least partially controlled bronchial asthma due to house dust mite allergy was examined in a study in children from the age of 5 [202] as well as in studies in children and adolescents from the age of 12 and adults [146, 179, 203, 204]. Statistically significant effects were confirmed for various endpoints such as asthma exacerbation [146], asthma control [203], impact on asthma medication consumption [179, 202], and symptom-medication score [204].

**Conclusion 20:** For adults, efficacy and safety of SLIT for allergic asthma caused by house dust mite allergy has been proven in studies. However, the clinical endpoints used in the studies differ significantly and therefore a comparison of the effect sizes as demonstrated in different studies is not possible. The data for children and adolescents are limited, although evidence exists for efficacy and safety of house dust mite SLIT in allergic asthma in this age group.

**Recommendation 6:** In patients with ARC clearly related to clinically relevant house dust mite sensitization, SCIT or SLIT should be performed using house dust mite extracts with confirmed efficacy and safety in clinical trials. In house dust mite-associated asthma, SCIT or SLIT may be considered to be applied. The prerequisite for this is at least partial asthma control, regardless of the level of therapy. This should be consistently re-evaluated during the course of therapy. At higher stages of asthma treatment regimes, AIT ought to be indicated and monitored by sufficiently experienced allergy pneumologists or pediatric pneumologists. (*strong consensus, agreement of 100%*)

### 4.7. Storage mite allergy 

Isolated sensitizations against storage mites (*Acarus siro*, *Tyrophagus putrescentiae* (Acaridae family), *Glycophagus domesticus*, *Lepidoglyphus destructor* (Glycyphagidae family)) are very rare in our region; usually there is a concomitant sensitization against house dust mites. Cross-reactivity between the storage mite and house dust mite allergens is low. However, cross reactions can exist among various species of storage mites. There is only very limited evidence for the efficacy of SCIT in ARC due to storage mite allergy [205] as for SLIT no studies could be identified.

**Conclusion 21:** Regarding preparations with storage mite extracts, only very little evidence for its clinical efficacy is available, and the availability of preparations for AIT is limited.

**Recommendation 7:** AIT with storage mite extracts may be considered to be applied in individual cases, even if no general recommendation can be given on the basis of clinical trials (see Chapter 3.3. keyword “*named-patient products”*). For the indication of AIT the clinical relevance of an identified allergic sensitization should be confirmed. (*strong consensus, agreement of 100%*)

### 4.8. Other allergens 

Many patients are sensitized against the above-mentioned allergens from birch, grasses, and house dust mites. With large regional variations, however, in more than 50% of patients also other inhalant allergens, such as tree pollen (ash, cypress, plane), herbal pollen (mugwort, ragweed, stinging nettle, ribwort plantain, pellitory), mammalian allergens, or mold spores (Alternaria sp. and Cladosporium sp.) play a role [206]. Due to the high grade of heterogeneity and the small numbers of patients, these allergens were excluded from the German TAO. This was taking into account foreseeable difficulties in enrolling large patient groups for high-quality studies in Germany within a realistic period of time. With few exceptions, studies on AIT preparations containing these allergens are small and not of high quality. 

**4.8.1. Ragweed (Ambrosia sp.)**

Ragweed is an exception within the rare allergens because it was recognized as one of the most significant inhalant allergens in North America more than 100 years ago [207]. *Ambrosia artemisiifolia* was introduced from North America to Eastern Europe more than a century ago as a bioinvader and, favored by climate change, is now spreading to northwestern Europe [208], resulting in increasing exposure to ragweed pollen, especially in eastern Austria but also in Germany [207, 209]. 

It is important to accurately differentiate between ragweed (*Ambrosia artemisiifolia*) and mugwort (*Artemisia vulgaris*), e.g., by component resolved diagnostics (CRD, major allergen components Amb a1 or Art v1, respectively), because these two herbs represent similar but only partially cross-reactive allergen sources, and most mugwort pollen-allergic patients would not benefit from AIT with ragweed-extract. In southwestern Germany, for example, extract-based diagnostics seems to suggest more frequent sensitization to ragweed (at 30%) than to mugwort (at 24%). However, in CRD detecting the primary allergen, true *Artemisia* sensitization clearly dominates with 13% of sera positive for Art v1 versus ragweed with only 2% sera positive for Amb a1 [210]. 

**4.8.1.1. Efficacy of AIT in ARC and allergy to ragweed pollen**

*SCIT*

There are no studies on SCIT with ragweed extracts in children. The efficacy of SCIT with ragweed pollen extracts in adults has been well confirmed in North American studies [211, 212]. 

*SLIT*

A ragweed tablet that has already been approved in North America for a decade has recently been approved in Germany and Austria. Evidence for clinical efficacy is high, but studies have largely been conducted in North America [213]. 

In a recent DBPC trial in North America and the EU, the efficacy of the ragweed tablet was impressively demonstrated with a 38% reduction in total combined score (TCS) (a combination of medication and symptom score) at the peak of the ragweed pollen season and 32% over the entire exposure period in 1,025 children with ARC and asthma [214]. Tolerability of the preparation was good. For other preparations, there is insufficient evidence of efficacy in children. 

In adult patients with ragweed allergy, a DBPC dose-finding study including 565 patients revealed a 21% improvement in TCS with half the dose and 27% improvement with the higher dose that has now gained marketing authorization [215, 216, 217]. 

**4.8.1.2. Efficacy of AIT in allergic asthma and allergy to ragweed pollen**

*SCIT*

There are no studies on SCIT with ragweed extracts in children. The above-mentioned older American study using a non-commercial extract in adult patients with allergic rhinitis also demonstrated an improvement of clinical symptoms in patients with concomitant allergic asthma [211]. 

*SLIT*

In the SLIT study reported above [214], 42% of children also suffered from bronchial asthma. In this subgroup, there was a 31% improvement in asthma symptoms in actively treated children versus placebo [214]. 

In a pivotal trial of the ragweed tablet, results in the subgroup of adult patients with bronchial asthma as treated with the finally approved dosage (19%) was reported, but the efficacy in asthma was not presented separately [216]. The rate of adverse events was low and did not differ from the whole population.

**Conclusion 22:** Efficacy of AIT in ragweed pollen allergy is well established for SCIT in adults with ARC and weakly established in asthma; data for children are lacking. For SLIT, the efficacy of a tablet preparation containing a ragweed pollen extract is very well demonstrated for adults and children with ARC.

**Recommendation 8:** In children and adults with proven clinically relevant ragweed allergy and rhinoconjunctivitis with/without bronchial asthma, SLIT with the ragweed pollen extract tablet should be performed. Alternatively, SCIT with a ragweed extract may be considered in adults with rhinitis and asthma. (*consensus, agreement of 87%*)

**4.8.2. Further allergens (Parietaria, pet allergens, mold spores)**

Pellitory (*Parietaria sp.*) is an important allergen source in the Mediterranean area, with a very long pollen season. In some regions, there is almost perennial allergen exposure [218, 219]. Although pellitory is also a member of the *Urticaceae* family, as is the native stinging nettle (*Urtica*), a cross-reaction between them seems to be unlikely [220]. Like stinging nettle, ribwort plantain (*Plantago sp*.), mugwort (*Artemisia sp*.), and ragweed (*Ambrosia*) are important allergen sources in mid and late summer in Central Europe [221]. True mugwort (*Artemisisa sp*.) pollen allergy is incidentally more common than ragweed pollen allergy in eastern Austria and southwestern Germany [206, 210]. 

*Pet allergens*

Many patients are sensitized to pets, and in many of them there is a strong desire for AIT rather than allergen avoidance by giving up keeping the pet. 

The development of a SCIT with synthetic Fel d1 peptides failed, after promising early studies [222], in phase III [223] and will not be available on the market. Also, an innovative approach by direct application of cat-allergens directly into the lymph node had not been developed any further [224]. 

Few studies have demonstrated some clinical efficacy of AIT in patients with allergies to cat or dog dander [225, 226, 227, 228, 229, 230]. Because of the limited evidence, AIT against cat dander requires critical risk and cost/benefit assessment on an individual, case-by-case decision. For dogs, evidence is insufficient, and the indication of AIT must be set even more critically here. Therefore, a Spanish consensus statement in 2018 concluded that allergen avoidance should always be preferred in the management of pet allergies. Only if this should be impossible, AIT may be considered in individual cases [231]. In the case of AIT with animal allergen extracts, particular attention must be paid to adequate control of any existing asthma during the course of AIT. 

More recent information (2020) on the clinical efficacy of AIT for cat and dog allergy derives from an experimental study investigating an alternative route of administration in terms of subcutaneous infusion using a syringe driver [232]. 

*Mold spores*

In practice, the question of mold allergy is often related to visible indoor mold infestation. The related hygienic and legal implications cannot be discussed here; the AWMF guideline for the medical clinical diagnostics of indoor mold exposure is helpful [233]. 

For allergy to spores of seasonal outdoor molds, evidence of clinical efficacy is limited to a few SCIT studies with *Alternaria alternata* and *Cladosporium herbarum* extracts [234, 235, 236]. A 3-year DBPC trial in children with *Alternaria* allergy demonstrated efficacy of SCIT beginning in the 2^nd^ year of treatment [237]. The difficulty in producing mold allergen extracts is that molds grown in a bioreactor produce differing allergens from the spores inhaled by patients. To overcome this problem, a multicenter Spanish study in 111 adolescents and adults shows promising results with SCIT using recombinant Alt a1 [238]. However, it will be some time before an approved compound will have been developed.

**Conclusion 23:** For the efficacy of AIT with other allergen extracts (except grasses, birch, mites, ragweed), a few studies for SCIT and SLIT are available but currently do not allow an evaluation of therapy efficacy.

**Recommendation 9:** AIT with other allergen extracts (except grasses, trees (*Betulacea*-like), house dust mites, ragweed) may be considered in individual cases, even if no general recommendations are possible due to the current clinical documentation (see Chapter 3.3 keyword *“named-patient products”*). Prior to AIT, the clinical relevance of an identified allergic sensitization and, especially in the case of pet dander allergies, the individual benefit/risk ratio should be carefully assessed. (*strong consensus, agreement of 100%*).

### 4.9. Peanut allergy 

At 19.0%, peanuts are, after insect stings (22.1%), the second most common reason for anaphylaxis in children and adolescents as shown in a 2016 evaluation based on the European Anaphylaxis Registry [239]. A more recent evaluation based on the same source carried out in 2020 reported a total of 10,624 cases of confirmed anaphylaxis since recording of these reactions started in 2006, of which 33.1% (n = 3,514) were anaphylaxis to food. Children and adolescents were involved in 1,962 of these cases. Of these 1,962 cases, 23.4% (n = 459) were induced by peanuts [240]. Immunological studies have shown that AIT with peanut leads to reduction of allergen-specific Th2 cells and IgE antibodies as well as production of allergen-blocking IgG and IgA antibodies [241, 242, 243, 244]. In contrast, there are divergent results regarding the induction of regulatory T cells indicating that these correspond to an only unstably differentiated variant of Th2 cells, which complicates a sustained tolerance induction over the duration of the therapy. 

According to a 2014 systematic review and meta-analysis, the prevalence in Europe for peanut allergy is 0.2% (95% CI 0.2 – 0.3%) when confirmed by provocation testing, and the prevalence is 1.6% (95% CI 1.2 – 1.9%) in case of a combination of history of peanut allergy and/or positive peanut provocation testing [245]. 

Spontaneous development of tolerance to peanut allergens is found in only about 20% of patients over a period of 3 years [246, 247, 248, 249]. Due to the potentially vital threat of anaphylaxis, the quality of life of patients and their families can be significantly reduced [250, 251]. 

In recent years, various approaches to AIT for the treatment of peanut allergy have been investigated in large clinical trials. In principle, there is the possibility of subcutaneous, sublingual, epicutaneous, and oral allergen application. 

The currently most advanced studies are in the area of oral tolerance induction (OTI). In this form of treatment, a precisely defined amount of a food allergen is swallowed daily in order to increase the tolerance threshold as prophylaxis of anaphylactic reactions after accidental exposure to small amounts of peanut. So far, this therapeutic approach has not been able to demonstrate any clinical benefit after cessation of the treatment-course in terms of disease modification. There are several clinical studies on efficacy and safety [252, 253]. One preparation was approved in 2021 for treatment of children and adolescents aged 4 – 17 years with confirmed peanut allergy. For this preparation, efficacy regarding the increase of the tolerance threshold was demonstrated. Furthermore, a partial improvement in the quality of life [253] could be shown. In a long-term study, the effects of therapy could be followed beyond the first year of continued treatment [254]. To date, no data are available regarding a therapy period of more than 24 months.

**Conclusion 24:** Various routes of application of AIT have been studied in peanut allergy, with the most clinical trials being available for oral tolerance induction (OTI). One OTI preparation has gained market authorization for the treatment of children and adolescents with a confirmed diagnosis of peanut allergy.

The indication for OIT with peanut should follow a special consideration of the expected therapy adherence as well as the level of suffering of the patients and their families. To avoid augmentation factors during therapy, special precautions should be taken into account due to the variety of therapy-related systemic side effects. Epinephrine autoinjectors must be kept at hand for the entire duration of therapy, including maintenance therapy. Initial up-dosing and the subsequent phase of dose escalation should be performed in centers experienced in the treatment of food allergy and anaphylaxis. It is currently being discussed in the expert committees, in which special group of 4- to 17-year-olds this therapy ought to be used preferentially.

**Recommendation 10:** Oral tolerance induction ought to be offered to children and adolescents with systemic peanut allergy after individualized consideration of the risk-benefit ratio with the prerequisite that both the initial up-dosing and the subsequent phase of dose escalation are performed in a center experienced in this indication. (*strong consensus, agreement of 100%*)

### 4.10. Atopic dermatitis 

**SCIT**

Clinical effects of AIT in patients with atopic dermatitis (AD) and proven sensitization to house dust mites were demonstrated in a 1-year study, with the SCORAD (Scoring Atopic Dermatitis) significantly improving under the therapy, which was administered at weekly intervals [255]. In a DBPC phase III trial using a polymerized house dust mite allergoid in 168 adults, a significant improvement in the SCORAD was only recorded in patients with severe AD [256]. An overall positive effect was found in a 2013 meta-analysis of the efficacy of AIT in AD, which included 8 randomized controlled studies (6 of which used SCIT and 2 used SLIT) [257]. In contrast, a Cochrane systematic review from 2016 [105] including 12 studies with a total of 733 patients showed no consistent effect of AIT with inhalant allergens in AD. 

**SLIT**

The efficacy of SLIT on AD has been investigated in few studies [258, 259, 260, 261], although the latter studies were not placebo-controlled. In a DBPC 4-arm study including a total of 239 patients, the SCORAD served as the primary endpoint. As reported in other studies, there was also a marked eczema improvement in the placebo group. However, when administered at high doses, there was a statistically significant effect compared to placebo in the FAS analysis after 6 weeks [258]. In a randomized parallel-group (phase IIIb) study in children and adolescents aged 5 – 18 years with house dust mite allergy and AD, a significantly reduced SCORAD was reported after 72 weeks of treatment using SLIT with a drop preparation [260].

**Conclusion 25:** No AIT preparation has gained market authorization for the treatment of AD. However, AD is not a contraindication to AIT if indicated otherwise. In most studies, SCIT and SLIT with house dust mite extracts have demonstrated positive effects in patients with AD related to house dust mite allergy.

**Recommendation 11:** If AIT is indicated for respiratory allergies, it should also be applied with AD as comorbidity. (*consensus, agreement of 92%*)

## Chapter 5. Indications and contraindications of AIT 

### 5.1. Background 

The indication, taking into account possible contraindications, is essential for the success of AIT. Asymptomatic allergic sensitization is not an indication for AIT. 

In the presence of ARC, there is only limited data on secondary prevention with regard to the development of allergic asthma, so that no clear recommendation can be currently given. However, real-life data confirm that AIT has a disease-modifying or preventive effect with regard to the aggravation of existing bronchial asthma [149] or the development of asthma symptoms [40, 41]. 

Various variables have an impact on therapy adherence and the overall success of AIT and therefore ought to be taken into account when planning therapy and, in particular, when selecting the route of administration. It is essential that patients are given detailed and careful information about the implementation of AIT. The primary objective at the beginning of AIT is a therapy duration of at least 3 years. 

### 5.2. Indication for AIT 

In addition to allergen-related symptoms with evidence of IgE-mediated sensitization, the prerequisites for an indication for AIT (Table 6) are the lack of sufficient allergen avoidance and the availability of suitable standardized or high-quality allergen extracts. In individual cases, the start of therapy may be indicated for patients younger than 5 years. This applies in particular to insect venom allergy if indicated and feasible.

**Conclusion 26:** Various prerequisites are essential for the indication to initiate AIT. Under certain conditions, AIT can also be used for patients with milder symptoms with the treatment goal of disease modification.

**Figure 3.**
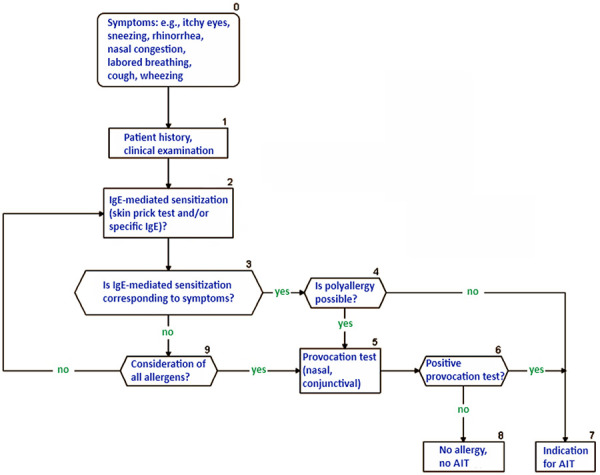
Diagnosis and indication of AIT (clinical algorithm) in moderate to severe rhinitis/rhinoconjunctivitis with/without asthma. If no clear correlation between the clinical symptoms (e.g., in the case of polysensitization to co-seasonal allergens) and the tested allergens is found, an organ-specific provocation test is indicated (Box 5). Since an allergic sensitization to competing allergens cannot be ruled out based on the history of the patients alone, nasal provocation with, e.g., house dust mite extract can be recommended to confirm the diagnosis before AIT particularly in the case of perennial symptoms. In the case of an allergy induced by animal dander, allergen avoidance is primarily indicated and AIT is only indicated in exceptional cases (see chapter above).

Figure 3 shows the clinical algorithm for adequate diagnosis and indication of AIT with seasonal and perennial allergens. Single or multiple symptoms may be relevant for assessing the severity of the disease. The individual disease burden is decisive for the indication for AIT [262]. Furthermore, criteria for examining the individual suitability of the patient for a certain AIT application route ought to be considered (Table 7), [33].

**Recommendation 12:** When establishing the indication for the start of AIT, certain prerequisites should be checked, and a clinical algorithm should be used. Criteria for examining the individual suitability of a patient for AIT should also be taken into account, and the patient should be involved in decision-making for patient-centered care through detailed information and education measures. (*strong consensus, agreement of 100%*)

When establishing the indication for AIT, it is also of central importance to provide the patient with comprehensive information in terms of patient-centered care and to discuss important points regarding the choice of the application route and the AIT product (Table 8).

**Conclusion 27:** Clear communication with the patient and family members regarding the application route and organizational precautions is essential for determining the therapy.

### 5.3. Component-based IgE diagnostics in AIT 

Component-based IgE diagnostics (or component resolved diagnostics (CRD)) can be helpful when polysensitization to pollen is present, especially with regard to sensitization to panallergens; it can also be used to assess the chances of success of AIT [263, 264] (Table 9). Patients without sensitization to major allergen components may have poorer therapeutic success with AIT in pollen allergy [265]. Sensitization to panallergens alone does not constitute an indication for AIT.

**Conclusion 28:** Component resolved diagnostics can be helpful in estimating the probability of success of AIT, particularly in the case of polysensitization.

### 5.4. Sensitization and clinical relevance 

In the case of perennial sensitization to house dust mites and animals, the clinical relevance of the sensitization must be determined in cases of doubt. Data on AIT in *Aspergillus* or *Penicillium* allergy are insufficient, which is why the treatment cannot be recommended. 

In the case of proven house dust mite allergy, AIT should be indicated and applied if mite avoidance measures (mite allergen-proof mattress covers (encasings), washable bed covers, and other measures to reduce house dust mite allergens) are not sufficiently effective. 

A meta-analysis published in 2008 questioned the efficacy of mite avoidance alone [266]. A significant reduction in house dust mites could only be documented in 17 of the 54 included studies. Overall, the intervention measures were heterogeneous, and subgroup analyzes in children were not presented. Due to the methodological deficits of this meta-analysis, the conclusion of the authors is not comprehensible. The intervention measures presented are therefore primarily indicated for patients with a clinically relevant house dust mite allergy [267, 268]. 

The German S3 guideline on allergy prevention also underlines the value of mite avoidance measures for tertiary prevention [269]. A recent, higher-quality, placebo-controlled study of mite-proof versus non-mite-proof covers also suggests that mite-proof encasings can significantly reduce the number of emergency room visits for asthma exacerbations in mite-allergic asthmatic children [270]. Before AIT with a house dust mite extract is initiated in children, nasal provocation is desirable, but not absolutely necessary. The professional societies in Austria do not stipulate any organ provocation before the indication for house dust mite AIT is made, even in adult patients. 

In the case of animal allergies and mold allergies, AIT is only indicated in certain cases (exception: *Alternaria* allergy, see below). 

When an animal allergy is present, allergen avoidance is the measure of choice. If this cannot be ensured, SCIT with animal allergen extracts is an option in certain cases (e.g., occupational exposure) (Figure 3). The greatest experience exists with AIT for cat allergies. 

While little evidence is available for perennial molds, and complete avoidance is often difficult, SCIT with mold allergens can be considered in the case of a seasonal mold allergy and well-characterized therapeutic allergens (*Alternaria, Cladosporium*) due to the slightly better evidence and the safety profile [234, 235, 237]. The main allergen of the mold *Alternaria alternata* (Alt a 1) is a major allergen similar to Bet v 1. Especially in eastern Austria, *Alternaria* is an important allergen with a long spore season from May to October. 

### 5.5. Mixtures of non-homologous allergen groups 

The efficacy of AIT depends not only on the selection of suitable patients, but also on the optimal therapeutic dose of each clinically relevant allergen and the duration of AIT (cumulative dose). The findings on the efficacy and immunological effects of AIT are mainly based on studies in which monotherapy with a single allergen extract was carried out. Different (non-homologous) allergen groups therefore ought not to be mixed in an allergen preparation, if the use of such a mixture is not supported by data from clinical trials. For example, in a DBPC SCIT trial, a chemically modified mixture of tree and grass pollen allergens showed significant (albeit moderate) clinical efficacy throughout the tree and grass pollen season in the 2^nd^ year of treatment [271]. 

Seasonal and perennial allergens are generally not mixed in one extract. This also applies to combinations of mite and animal allergens, mite and mold allergens, or extracts with pollen and mold allergens, which must never be mixed due to enzymatic degradation processes [272].

**Recommendation 13:** Seasonal and perennial allergens should never be mixed in one extract. In addition, combinations of mite and animal allergens, mite and mold allergens, or pollen and mold allergens, should never be mixed due to enzymatic degradation processes. (*strong consensus, agreement of 100%*)

### 5.6. Contraindications for AIT 

A number of contraindications must be considered when deciding on SCIT and SLIT [33, 273] (Table 10). 

Uncontrolled asthma is a risk factor for systemic side effects of AIT. It is therefore recommended not to use AIT (both SCIT and SLIT) in patients with uncontrolled or severe asthma or in adult asthma patients with markedly impaired lung function (FEV_1_ ≤ 70% predicted value) [141]. Uncontrolled bronchial asthma is also a contraindication for initiating AIT in children and adolescents [92, 275]. First of all, better asthma symptom control should be aimed for by, among other things, intensifying the therapy, in order to then be able to start the causal therapy. If asthma exacerbates under AIT, asthma therapy ought to be consistently escalated and AIT should be paused until control is regained [276]. 

Although pregnancy is considered a contraindication for the start of AIT, continuation of SCIT is advisable in the case of a life-threatening allergy to insect venom (bee, wasp) and good tolerability, and continuation of AIT with aeroallergens is possible if tolerability is good (according to the recommendations of the SmPC and package leaflet) [277, 278]. Only in very rare cases, SCIT can be initiated during pregnancy (e.g., in the case of a life-threatening insect venom allergy) [93]. 

Medication with β-blockers (also when applied locally, such as ophthalmics) is sometimes listed as a contraindication under SCIT in the SmPC characteristics but is now only to be regarded as a relative contraindication in the case of insect venom allergy [276]. A large international multicenter trial showed no increase in the risk of side effects either with treatment with β-blockers or ACE inhibitors [279]. The same presumably also applies to AT-II blockers that interfere with the renin-angiotensin metabolism. When establishing the indication for AIT, a detailed risk-benefit analysis ought to be carried out together with the patient after careful explanation. The decision about continuing the possibly necessary therapy with these substances must be made together with the prescribing physician on an individual basis. Even if there are no specific data, therapy with immunosuppressives suggests a weakening of the effect of AIT (Table 10) [280]. 

With clear, strictly defined indications for SCIT (severe anaphylactic reactions to insect venom in the medical history), a Swiss case series of 25 patients with heart disease and a clear indication for taking β-blockers reported no increase in severe side effects under SCIT [281]. 

Indications and contraindications (Table 6, Table 10) must also be taken into account for the sublingual application of AIT [33, 92, 98]. Systemic side effects are observed less frequently with SLIT than with SCIT [33, 99]. Patients with a chronic or very frequently recurring disease of the oral mucosa (e.g., chronic recurrent aphthae) are not suitable for SLIT. With modern, effective high-dose SLIT therapies, especially with tablets, an increase in the occurrence or reactivation of an eosinophilic esophagitis, which is rare in itself, has been observed [282, 283, 284, 285, 286, 287]. Therefore, a history of these gastrointestinal disorders ought to be considered a contraindication for SLIT. Otherwise, the contraindications for SLIT are similar to those for SCIT (Table 10), whereby the SmPC and package leaflet of the used product must be taken into account. In younger children in particular, it is important to check before starting SLIT whether correct sublingual application and appropriate contact time with the mucosa are realizable. 

In the case of severe psychiatric disorders, the indication and the route of application ought to be weighed very carefully [33]. 

Structural requirements and medical knowledge, skills, and abilities have a major impact on the general safety of AIT [288]. This argues for treating patients with risk factors only in centers with sufficient experience in the use of AIT.

**Conclusion 29:** Various contraindications are found for AIT, with only very minor differences between the application routes.

**Recommendation 14:** Various contraindications speak against the use of AIT and should be taken into account. When assessing those, the respective summary of product characteristics and package leaflet should generally be taken into account. In justified individual cases, AIT is possible after weighing the risk/benefit-ratio, even if the contraindications mentioned are present. (*strong consensus, agreement of 100%*)

### 5.7. AIT despite contraindications 

In selected cases, AIT can also be initiated if there are relative contraindications. Well-controlled Hashimoto’s autoimmune thyroiditis is a typical example of an autoimmune disease that can be easily compensated for with medication, and this type of disease is not necessarily a contraindication for AIT. In other autoimmune diseases such as multiple sclerosis, myasthenia gravis, lupus erythematosus, rheumatoid arthritis, or Crohn’s disease, AIT can be initiated after individual consideration, taking into account disease activity and course. Evidence for AIT triggering autoimmune diseases is based on case descriptions (15 articles including 22 cases, 12 of which with vasculitis) [289]. Evidence for assessing AIT in nephrotic syndrome is similarly insufficient. 

A registry-based observational study from Denmark showed that over a 10-year observation period (1997 – 2006), SCIT was associated with a lower mortality (hazard ratio (HR) 0.71; 95% CI 0.62 – 0.81) as well as a lower incidence of myocardial infarction (HR 0.70; 95% CI 0.52 – 0.93) and autoimmune disease (HR 0.86; 95% CI 0 .74 – 0.99) [290]. 

The risk of developing an autoimmune disease from SCIT is therefore probably very low, but ought to be taken into account, especially since it is a multi-year treatment. If there is a suspicion, AIT ought to be discontinued until the association has been clarified. 

A special case among the contraindications in immunodeficiency listed in Table 10 is acquired immunodeficiency in chronic, stable HIV (human immunodeficiency virus) infection with negative HIV replication and normal CD4 counts that is well controlled with combined antiretroviral therapy (cART). So far, case reports and a small case series of 3 patients have been reported in the literature [291, 292]. 

AIT under cART appears to be safe, non-aggravating, and effective [293]. If there is a clear indication, AIT can be started in HIV-positive patients on cART and a stable disease state. 

Since old age is not a contraindication for AIT and the incidence of tumor diseases increases with age, there is an increasing population of patients with ARC/asthma who have a history of oncological disease. A relatively recent tumor disease that is currently stable does not necessarily represent a contraindication. AIT could be completed in a case series of 4 patients with melanoma and insect venom allergy and 1 patient with ARC and breast cancer. In most of these patients, no tumor reactivation could be observed after even more than 5 years of follow-up [294].

**Conclusion 30:** When establishing the indication, factors that may impact the clinical efficacy must be taken into account. If there is an indication, and after considering comorbidities, AIT is also possible in patients over 65 years of age or in patients with autoimmune disease. Differences between SCIT and SLIT are primarily to be considered with regard to contraindications. Even if contraindications are present, there may be an indication for AIT in justified individual cases.

**Recommendation 15:** Various variables impact the success of AIT and therefore ought to be taken into account when planning therapy. (*strong consensus, agreement of 100%*)

## Chapter 6. AIT administration 

### 6.1. Concomitant pharmacological therapy 

AIT in adults is indicated for moderate to severe intermittent and persistent allergic rhinitis/rhinoconjunctivitis and/or at least partially controlled allergic asthma when allergen avoidance or pharmacotherapy cannot adequately control symptoms. In children, AIT can be indicated even if only mild symptoms are present in order to benefit from its potentially preventive/disease-modifying effect. In both cases, pharmacotherapy is the basis of therapy for both ARC and asthma, and AIT is used in addition to it. The pharmacotherapy should be administered according to the guidelines for the indications ARC [295] or asthma [141, 142], respectively and is based on the use of systemic antihistamines and/or topical steroids (also combined with topical antihistamines) in ARC, and therapy with inhaled corticosteroids alone or in combination with long-acting β-2-agonists (LABA), leukotriene receptor antagonists (LTRA), or long-acting muscarinic receptor antagonists (LAMA) in asthma. The simultaneous use of this pharmacotherapy with AIT has no effect on the efficacy of AIT but can have a beneficial effect on or alleviate the side effects of AIT due to the anti-allergic or anti-inflammatory effect of the active ingredients used. Intramuscular injections of a depot corticosteroid, which are unfortunately still used, must be warned of as this leads to significant local and systemic side effects and is not part of a guideline-based pharmacotherapy [296]. The side effects are common and sometimes serious. For example, lipodystrophy, muscle atrophy, abscesses, and femoral head necrosis can occur. In the long term, there is a risk of diabetes, osteoporosis, and cataracts [297, 298, 299, 300]. Patients should not associate these injections with AIT or even confuse them with AIT. There are no studies that have examined this form of treatment and proven it to be safe and effective.

**Conclusion 31:** Pharmacotherapy is the basis of therapy for both ARC and asthma, and AIT is applied in addition to it.

**Recommendation 16:** AIT should be applied in addition to guideline-based pharmacotherapy of ARC or allergic asthma. An intramuscular injection of a depot corticosteroid is not part of a guideline-based pharmacotherapy and should not be carried out. (*strong consensus, agreement of 100%*)

### 6.2. Prescription and administration of AIT 

Since January 1, 1996, the SmPC and package leaflet of the therapeutic allergens used in Germany must contain the following warning: “*Hyposensitization vaccines for injection may only be prescribed and administered by physicians trained or experienced in allergy*” (PEI, communication dated April 5, 1995). 

In Austria, therapeutic allergens may be prescribed and AIT applied by specialists with expertise in allergy. The maintenance therapy of AIT can be delegated to a general practitioner. Furthermore, in Austria, medical tasks may be delegated to medical health professionals subordinate to the delegating physician and bound by instructions. However, the responsibility remains with the delegating physician. 

§49(3) Austrian Medical Act: *“(3) In individual cases, medical doctors may delegate medical acts to other health professionals or to trainees of a health profession, provided that the respective act is covered by the professional competence of the latter (Fed Law Nr I 2001/110). The medical doctor is responsible for this direction. […]”* [301].

**Conclusion 32:** In Germany, Austria, and Switzerland, different legal provisions apply to physicians applying AIT.

**Recommendation 17:** AIT should be performed by physicians who either have the sub-specialization in allergy (“(Zusatz-)weiterbildung Allergologie”, Germany), specialization in allergy (“Spezialisierung in Allergologie”, Austria), or are allergy specialists (“Facharzt für Allergologie”, Switzerland), or have sufficient experience with this therapy and are capable to providing emergency treatment for adverse drug reactions (anaphylactic shock, severe asthma attack, etc.). Patient information including documentation should be carried out before initiating AIT. (*strong consensus, agreement of 100%*)

In Switzerland, AIT can also be carried out by primary care providers, provided that an allergy workup has taken place beforehand. 

If AIT is performed or continued by another physician after the indication has been established, close cooperation is required to ensure consistent and low-risk administration of AIT. This applies in particular to the occurrence of adverse drug reactions (ADR). If necessary, the patient should be referred back to the physician who initially indicated AIT.

**Recommendation 18:** If AIT does not grant any noticeable success after 1 or 2 years at the latest, it should be critically reviewed by a physician meeting the above criteria (*Recommendation 17*). (*strong consensus, agreement of 100%*)

If necessary, a change of the preparation or of the route of application, a change from a pre-seasonal to a perennial treatment regimen, or even a discontinuation of therapy can be considered. In general, the administration of SCIT and SLIT is only recommended with preparations for which there has been sufficient evidence of safety and efficacy in clinical trials (see Chapter 4). In the case of rare allergens, the possibilities and limitations of the feasibility of clinical trials must be taken into account due to the small patient collectives. The German TAO can serve as a guide for this. 

### 6.3. Patient information 

Before initiating AIT, patients must be informed about the implementation, type and duration of treatment, the expected effects, possible risks, and possible alternatives. The Medical Association of German Allergologists (Ärzteverband Deutscher Allergologen (AeDA)) has issued a comprehensive statement on this ([302]; download at: www.aeda.de). This risk information is also referred to as self-determination information and must take place before consent to a medical measure. According to the provision of German Civil Code Section 630e paragraph 1 sentences 1 and 2, the *“… treating party is obliged to inform the patient of all and any circumstances which are relevant to consent. This includes in particular the nature, extent, implementation, anticipated consequences and risks involved in the measure, as well as its necessity, urgency, suitability and prospects for success with regard to the diagnosis or the therapy.”*

If treatment contains several different essential risks, the patient must be informed about all of these risks. It is important to note that this information must always be given in oral form, and text documents can only be used as a supplement. Section 630e paragraph 2, number 1, 2^nd^ clause: *“… additionally, documents may also be referred to which the patient receives in text form ...”.* However, the oral information is always decisive. The patient must receive the supplementary text (Section 630e, paragraph 2): *“The patient shall be provided with duplicates of documents which he/she has signed in connection with the information or consent.”* According to Section 630h (paragraph 2, sentence 1) the physician has the obligation to provide evidence that the patient was adequately informed and has consented to the treatment: *“The treating party is to prove that he/she has acquired consent in accordance with section 630d and provided information in accordance with the requirements of section 630e.”*

**Figure 4.**
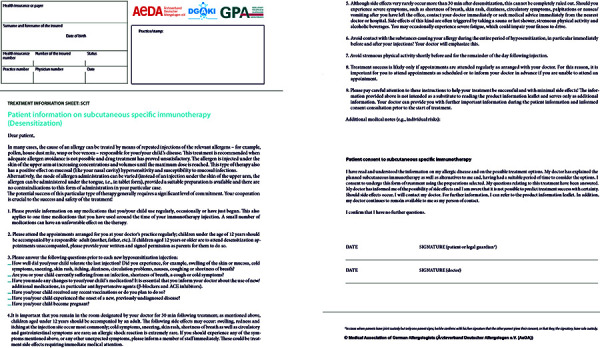
Patient information on subcutaneous specific immunotherapy (Desensitization).

**Figure 5.**
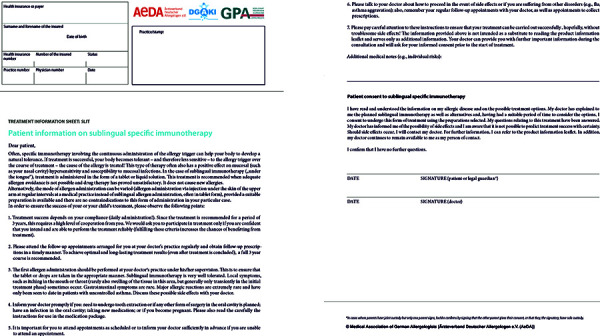
Patient information on sublingual specific immunotherapy.

It is recommended to document the handing of the text in the patient file. AeDA has provided this information in text form in English language (“*Treatment Information Sheet SCIT” *(Figure 4)* and “Treatment Information Sheet SLIT” *(Figure 5)*,* download from: www.aeda.de). Patient information must be given in an understandable way (Secton 630e, paragraph 2, number 2) *and “... be provided in good time so that the patient can take his/her decision on consent in a well-considered manner”*.

**Conclusion 33:** A detailed and well-documented patient information is a prerequisite for the implementation of AIT. The documents ’Treatment Information Sheet SCIT’ and ‘Treatment Information Sheet SLIT’ can be helpful in this regard.

Patient information can be delegated only to physicians who meet the above-mentioned criteria (recommendation 17), not to non-medical (specialist) personnel. The aim of informing the patient about therapy alternatives should be that the patient is able to make a choice between several treatment alternatives. However, this does not mean that the treating physician has to offer all treatment alternatives. Adequate documentation of the information given in oral form is mandatory, and a written consent of the patient or the parents/legal guardians is recommended. 

### 6.4. Compliance and adherence 

The term “*compliance*” means that the patient passively follows the physician’s instructions, which means that patients are primarily responsible for the success or failure of a therapy. The more modern concept of “*adherence*”, on the other hand, means that patient and physician have made the therapy decision together, i.e., they have both set the goals of therapy and decided on how to achieve them [303, 304, 305, 306]. 

For AIT, implementation of the medical recommendations is particularly important since the success depends on the duration of an adequate therapy. Analogous to other treatments, comprehensive information provided to the patient about the mode of action of the AIT also improves the patient’s therapy adherence [33, 307, 308, 309, 310]. 

As SCIT is applied by the physician, monitoring of therapy adherence is generally easier than for SLIT. The extent to which this results in better therapy adherence to SCIT compared to SLIT is currently controversial, because it significantly depends on whether adherence was examined under *“real-life”* conditions or in the context of clinical studies. In the latter, adherence is, understandably, better [305]. 

Regarding adherence, data from clinical trials on SCIT and SLIT were compiled [311], and a therapy adherence of ~ 70% for SCIT and 75% for SLIT has been reported. However, these results are limited because studies from the United States and Europe using different therapy regimens and indications as well as patient groups were combined. In a randomized study, 271 patients (aged 15 – 65 years) with allergic rhinitis with or without concomitant asthma received SLIT over a period of 3 years [139]. In almost 72% of patients treated over the entire period, the authors found an adherence rate of more than 80%, and in 18% of patients, the adherence rate was between 60 and 80% [139]. Another review [312] found no differences in the adherence rates between SCIT and SLIT; the rates found in recent studies are between 75 and 90%, regardless of the route of application. However, these data also come from clinical trials and thus reflect the therapy adherence in everyday care [125] very imprecisely. 

Lack of therapy adherence threatens treatment success. This is the conclusion of an analysis of real statutory health insurance SCIT prescription data [313], which showed a decreasing persistence rate over the years: In only 24% of the patients treated with common SCIT products, SCIT was still performed in the 3^rd^ year. Data from Germany and Italy published as a “poster” and a “letter”, respectively, show similar negative results for SLIT over 3 years (13.2 – 22.7%) [311, 314]. A further analysis of real statutory health insurance prescription data determined the persistence of 1,409 patients treated with market-leading SCIT and SLIT products [315]. This analysis identified unsatisfactory persistence rates in the 3^rd^ year of therapy ranging from 34 to 51% of patients. An analysis of statutory health insurance prescription data for 562 children and adolescents aged 4 – 18 years showed a persistence rate of 44.1% for AIT in the 3^rd^ year [316]. Recent studies under RWE conditions show significant and also age-dependent differences between adherence to SCIT and SLIT products. For example, a retrospective study based on prescription data showed 38.6% adherence to a house dust mite allergoid after 3 years of subcutaneous therapy; significantly higher adherence rates were seen in the children studied, at 47.9% compared with 41.3% in adolescents and 35.5% in adults [178]. 

A retrospective RWE study, also based on prescription data of treatment adherence with SLIT and SCIT preparations (grass and tree pollen), revealed equally much lower adherence rates with significant differences between the two forms of application: while SCIT adherence to grass and tree pollen allergoid was 37.5% and 35.0%, respectively, after 3 years, SLIT adherence to the two drop and tablet preparations studied ranged from 9.6 to 13.4% for the grass pollen and 10.3 to 18.2% for the tree pollen preparations. Again, adherence of treated children was significantly better than that of adults for subcutaneous tree pollen and grass pollen allergoids [39]. A retrospective cohort analysis from Germany based on prescription data of 5,677 patients receiving SCIT with a house dust mite allergoid and 4,720 patients receiving SLIT with a house dust mite tablet showed a therapy adherence of 55.0% to SCIT after 3 years, whereas adherence to SLIT was 30.3% [317]. Significant differences between the two treatment forms could also be demonstrated in another study showing 42% adherence over 3 years to pollen SCIT and 45% to house dust mite SCIT, compared with 16% to pollen SLIT [318]. 

In another study, SCIT and SLIT patients were asked, among other things, about adverse aspects of treatment after completion of therapy [304]. The results may provide insight into reasons for unsatisfactory treatment adherence. 69.5% of the patients complained about the time-consuming nature of the therapy and 62.5% about the side effects. 60.7% of patients felt no relief of symptoms and 53.7% received insufficient information about the treatment. Accordingly, inadequate patient information as well as inadequate implementation of therapy and practice management represent important reasons for premature discontinuation of therapy. From this, reasonable recommendations for increasing treatment adherence can be derived (Table 11). 

Improving adherence in AIT is one of the most important future tasks to ensure efficacy of this causal treatment option. Additional incentives and supports for physician performance (such as the Bavarian selective contract) are desirable.

**Conclusion 34:** Irrespective of the mode of administration, therapy adherence of AIT patients is lower than assumed by physicians, although it is of decisive importance for treatment success. Improving adherence to AIT is one of the most important tasks to ensure optimal effectiveness of the treatment. Consideration of patient-specific requirements, comprehensive instruction of the patients, and optimized practice/clinic management are decisive for a high degree of adherence.

### 6.5. Subcutaneous immunotherapy 

Table 12 gives an overview of recommendable procedures when performing SCIT. 

Before the injection, the patient is asked about relevant current symptoms (e.g., allergic symptoms or signs of infection), tolerability of the last injection, past illnesses, new or changed medication intake, and vaccinations, and the interval from the last injection is checked [321]. The therapy allergen preparations are to be stored in the refrigerator at ~ 4 °C. Confusion can be prevented, for example, by reading the preparation’s and the patient’s name aloud in the presence of the patient. 

The injection has to be carried out by a physician using a 1-mL syringe with fine graduations of down to 0.1 mL with an injection needle (size 14 – 18 gauge, short bevel, needle of sufficient length) after disinfecting the relevant skin area. The injections are made strictly subcutaneously in a lifted skin fold after prior aspiration (depending on the injection volume several times) a hand’s width above the olecranon on the extensor side of the upper arms and are documented, stating the injection site and the dose. When selecting the injection needle, the Biological Substances Ordinance (Biostoffverordnung) BGR 250/TBR 250 (professional association rules / technical rules for biological agents in health care and welfare), the national guideline for avoiding injuries from sharp/pointed instruments in the hospital and health sector, must be followed in Germany, and where appropriate an injection needle with an injury-proof injection system (retraction system, needle shield, or similar) has to be chosen [322]. 

After the injection, the patient must stay under medical supervision for at least 30 minutes [33]. 

The patient ought to immediately inform the staff of any symptoms indicating an allergic reaction during this time and afterwards. The injection site has to be checked after the waiting time. In the event of an increased local reaction, the diameter must be documented, since the dose may have to be adjusted for the next injection in accordance with the respective instructions given in the SmPC and package leaflet of the SCIT therapy allergen extract used.

**Conclusion 35:** Various precautions and procedures are helpful to ensure that SCIT is performed in accordance with the guidelines. These can also be found in the summary of product characteristics and package leaflet of each SCIT preparation.

**Recommendation 19: **The summary of product characteristics and package leaflet of the SCIT preparations must be followed. When SCIT is used, augmentation factors for allergic reactions (e.g., physical exertion, visits to the sauna, alcohol consumption) should be avoided shortly before and for the rest of the day after the injection. The interval between a SCIT injection and a planned vaccination ought to be at least 1 week. It is therefore recommended to apply vaccinations in the maintenance phase of SCIT. Immediate vaccinations (e.g., tetanus after injuries) may be considered at any time. SCIT is then continued in accordance with the summary of product characteristics and package leaflet. (*strong consensus, agreement of 100%*)

**Recommendation 20:** In general, the administration of both SCIT and SLIT is only recommended with preparations for which there has been sufficient evidence of safety and efficacy in clinical studies. Exceptions apply to rare allergens (those that are not included in the German Therapy Allergen Ordinance) for which sufficiently large studies cannot be carried out due to a lack of patients. (*strong consensus, agreement of 100%*)

For many AIT preparations/therapy allergens, therapy consists of initial treatment (initial, dose escalation phase) in which the dose is gradually increased, and subsequent continuation treatment (maintenance phase). All dosing regimens are manufacturer- and preparation-related recommendations that must be individually adjusted. Various initial updosing schemes are available for dose escalation, which can be generalized as conventional updosing, cluster updosing, and rush updosing. 

For seasonal aeroallergens, it is generally recommended that therapy should be escalated to the maximum dose outside the allergy season and continued for at least three additional years [1, 33]. For certain therapy allergens, intraseasonal start of therapy is also possible (according to the SmPC and package leaflet) [323]. Therapy intervals range from 3 to 7 days for aqueous therapy allergens and from 1 to 2 weeks for semi-depot therapy allergens during the dose escalation phase (often doubling of the previous dose according to the SmPC and package leaflet). In the case of aeroallergens, semi-depot therapy allergens are predominantly used for SCIT. In cluster or rush dose escalation regimens, multiple injections are given per treatment day [324, 325]. 

A co-seasonally performed SCIT (continuation during the symptom season) without dose reduction is possible if the SmPC and package leaflet allow for it, if no allergic symptoms are present at the time of injection, and if careful clinical documentation is made. Due to potentially different biological activity (preparation- and manufacturer-specific SmPC and package leaflet) at the start of a new batch, a reduction in the planned dose may also be necessary when treatment is continued. 

If the injection interval is exceeded, the dose is reduced in accordance with the SmPC and package leaflet, and the longer the interval is exceeded, the more so. In the case of respiratory allergies, SCIT ought to last 3 years at least. 

Although there are only a few controlled studies for parallel AIT with two different allergen extracts in the same session [326], it has proven useful in everyday clinical practice to leave a gap of (15 to) 30 minutes between the injections or sublingual applications for safety reasons. However, the SmPC and package leaflet should be referred to. After the last injection, the usual medical supervision time of 30 minutes must be observed. Thus, several complete therapy sessions (application, 30-minutes follow-up) are possible on 1 day with several preparations. Alternatively, those can be administered on different days. 

AIT is generally applied in an outpatient setting. If a rush updosing scheme is used (see below) or in patients at risk (more pronounced general reactions, relative contraindications), it ought to be considered to initiate SCIT in an inpatient setting. If required from a medical point of view, respiratory and cardiovascular monitoring ought to be carried out in patients with an increased risk, and it is recommended to carry out lung function tests in patients with allergic asthma at regular intervals, and before and after each injection, if indicated. 

For information on the indications, contraindications, options for treatment monitoring, and duration of therapy in Hymenoptera venom AIT, it is referred to the AWMF guideline on diagnosis and therapy of bee and wasp venom allergy [327]. 

### 6.6. Sublingual immunotherapy 

SLIT is performed in an outpatient setting in accordance with the manufacturer’s SmPC and package leaflet. 

Depending on the preparation and the manufacturer’s instructions, the first dose should be taken under the supervision and follow-up of an allergologically experienced physician. According to the respective preparation- and manufacturer-specific information (SmPC and package leaflet), SLIT with some therapy allergens can be initiated during the pollen season (“intra-seasonal start”). 

In the case of viral infections of the respiratory tract, the application can either be continued according to the physician’s recommendation or it must be interrupted (refer to SmPC and package leaflet). In the case of acute inflammation or injuries of the mucous membranes of the mouth/pharynx, major oropharyngeal or dental surgery, acute gastroenteritis, or uncontrolled asthma, no allergen extract ought to be taken (refer to SmPC and package leaflet). For a mite SLIT preparation, the safety of AIT has also been proven in patients with uncontrolled but non-severe asthma [92, 146].

**Conclusion 36:** Various precautions and procedures are helpful to ensure that SLIT is performed in accordance with the guidelines. These are based on the recommendations in the summary of product characteristics and package leaflet of the SLIT preparations.

Co-seasonal SLIT (continuation during the symptom season) without dose reduction is possible if the SmPC and package leaflet allow for it, if allergic symptoms are absent or only minor, and if clinical documentation is carried out carefully. The duration of SLIT ought to be at least be 3 years. If the treatment is continued in another practice, there ought to be close co-operation with the physician who originally established the indication, especially with regard to safety and effectiveness.

**Recommendation 21:** SLIT ought to be performed in accordance with the manufacturer’s summary of product characteristics and package leaflet. If AIT is applied or continued by another physician after the indication has been established, close cooperation is required to ensure a consistent and low-risk treatment. (*strong consensus, agreement of 100%*)

## Chapter 7. Safety, risk factors, and adverse events 

### 7.1. SCIT 

AIT with SCIT preparations is safe and well tolerated, if it is applied correctly, if patient selection is based on the indication, and if it is performed in a practice/clinic experienced with this therapy [328, 329, 330]. Local reactions at the injection site (redness, swelling, itching) are very common but can be easily treated locally (e.g., cooling or topical glucocorticoids) or by systemic antihistamines. 

If increased local reactions (redness and/or swelling > 10 cm in diameter) occur at the injection site, the specific manufacturer’s information and package leaflet of the respective SCIT preparation must be taken into account for the dosage of the following injection. However, an American working group was able to show in a retrospective analysis of their own patient data that increased local reactions do not represent an increased individual risk for the occurrence of systemic reactions [331]. 

In the case of Al(OH)-containing SCIT products, persistent nodules or granulomas can rarely occur, particularly if an incorrect intradermal instead of subcutaneous application technique was used, and can be regarded possibly as an expression of an Al(OH) contact allergy and most likely as a foreign body reaction [332, 333, 334]. In such cases, it is advisable to switch to an allergen extract that does not contain Al(OH). Aluminium salts are the most commonly used depot adjuvants in allergen-specific SCIT [335]. Risks from aluminium as an adjuvant have been discussed critically for a long time. A statement on the safety of aluminium in therapy allergens published by the PEI in 2014 addresses local tolerability, sensitization potential, toxicity, and the data from pharmacovigilance in Germany [336]. 

According to this, the sensitization potential of aluminium can be assumed to be generally low, and there have only been isolated reports of sensitization in patients when using SCIT [332, 333, 337]. Toxic effects depend on the amount of aluminium resorbed [337]. The contribution of SCIT – assuming 3 years of therapy, based on a maintenance dose of 8 subcutaneous injections per year with 0.5 mg aluminium each – to the lifelong accumulation of aluminium in the organism is to be classified as low compared to other sources. The specific evaluation of all reported side effects of therapy allergens from 1986 to 2013 did not result in a safety signal either. The PEI concludes that the currently available scientific data do not suggest a risk to children or adults from SCIT with aluminium-adjuvanted allergens, and thus, based on current knowledge, there are no reasons to change the practice of using approved therapy allergens adjuvanted with aluminium. 

To further improve the data on the safety of aluminium-containing adjuvants, a research program on a physiologically based toxicokinetics (PBTK) model was initiated and funded by the PEI in 2015 to assist them in continuously assessing the safety of aluminium-adjuvanted drugs [338].

**Conclusion 37:** Based on current knowledge, the use of aluminium-adjuvanted SCIT preparations does not pose a risk of toxic effects in children or adults.

Possible systemic allergic reactions in SCIT can include mild to severe forms of skin, gastrointestinal, respiratory, or cardiovascular reactions. In a survey conducted between 2008 and 2013 by the American Academy of Allergy, Asthma & Immunology (AAAAI) and the American College of Asthma, Allergy and Immunology (ACAAI) based on 28.9 million injection visits in 344,480 patients, systemic reactions were found in a total of 1.9% of patients, including grade 3 reactions (WAO classification) in 0.49/10,000 injections (0.08% of patients) and grade 4 reactions in 63 cases (0.02% of patients) [339, 340]. 14% of the systemic reactions started more than 30 minutes after the injection, but these were mostly mild to moderate, and none was fatal. Two confirmed fatal reactions have been reported in patients treated by allergists and 2 more in patients treated by non-allergists [340, 341, 342]. In the continuation of the survey, 5 additional confirmed deaths were reported from 2015 to 2017, corresponding to 0.8 fatal reactions per year over a total period of 9 years [343]. 

The European Survey on Adverse Systemic Reactions in Allergen Immunotherapy (EASSI), conducted in France, Germany, and Spain in 2012 – 2014, revealed 97 systemic reactions in the context of 3,398 SCIT therapies with 57,463 SCIT doses using MedDRA terminology (www.medra.org), i.e., systemic reactions occurred in 2.9% of all treatments, and in 0.17% of all injections, respectively. Fatal reactions did not occur [344]. In the subgroup of children and adolescents (11.7 ± 3.9 years), systemic reactions occurred in 1.53% of patients receiving 1,127 treatments with 19,669 injections [345]. In the overall group, use of unmodified extracts, non-use of symptomatic allergy medications (as a possible indication of unsatisfactory symptom control), asthma, sensitization to animals or pollen, cluster versus rush schemes, and previous episodes of anaphylaxis were associated with a higher risk of systemic reactions. 

In another prospective observational study carried out from 2012 – 2014 in 581 pediatric patients, immediate-type systemic reactions were seen in 2.2% of patients receiving a total of 10,015 injections, and delayed-type systemic reactions occurred in 7.4% of patients [346]. Severe systemic reactions of grade III according the Ring and Messmer classification [347] were observed in 0.03% of all treatments, all occurring within 30 minutes of injection. No grade IV reactions were seen [346]. 

According to PEI data (1991 – 2000), the incidence of severe reactions was calculated to be 0.002 – 0.0076% (based on the number of injections) for non-modified (native) allergen extracts and 0.0005 – 0.01% for chemically modified allergen extracts (allergoids) [348]. Severe reactions can sometimes be explained by risk factors and can usually be avoided by caution and prophylaxis [328, 330, 349]. Table 13 provides an overview of possible risk factors that may be associated with the occurrence of systemic reactions in AIT.

**Conclusion 38:** Most adverse reactions are mild to moderate in severity and are easy to manage. The occurrence of severe, potentially life-threatening systemic reactions in SCIT is possible but very rare if all safety measures are observed.

**Recommendation 22:** After the occurrence of a severe reaction in the context of AIT, the decision on the continuation or discontinuation of the therapy should be made by an allergist or a physician experienced in this therapy, taking into account the risks of continuing the therapy, the indication, and the therapy alternatives as shared decision making with the patient (see also Chapter 5 and 6). (*strong consensus, agreement of 100%*)

For this purpose, the patient may need to be referred to the physician who originally established the indication for SCIT. The risk factors described above ought to be identified and avoided in the future in connection with SCIT. In case of continuation of the therapy, a dose reduction is recommended according to the SmPC and package leaflet of the respective preparation. In case of adverse reactions, premedication with an oral histamine 1 (H1) receptor antagonist is possible to reduce the frequency and severity of possible systemic reactions. However, these cannot be excluded despite premedication [280, 324, 325, 352, 353, 354]. Comprehensive patient education at the beginning of therapy is of particular importance (see also Chapter 6). The management of severe adverse effects is described in detail in Chapter 8 (Emergency treatment). 

### 7.2. SLIT 

AIT with SLIT preparations is classified as safe when applied appropriately, with indication-specific patient selection and implementation [125, 355]. Side effects are dose-dependent [183, 356, 357] and have been reported in reviews to range from ~ 45 to 80% [358, 359]. Side effects are usually mild and occur predominantly as local mucosal reactions (oropharyngeal pruritus, paresthesia, oral/pharyngeal swelling, ulceration of the tongue, etc.) [125, 183, 350, 356, 357, 358, 359, 360, 361]. Local adverse drug reactions manifest predominantly in the initiation phase or during the first weeks of treatment [362] and are usually self-limiting after a few weeks of therapy [125]. However, they represent a relevant risk factor for premature discontinuation of therapy, especially in the initiation phase. Therefore, comprehensive patient information at the beginning of the therapy is of particular importance (see also Chapter 6, keyword “patient information”). Gastrointestinal symptoms during SLIT have been reported in 14% of cases [355]. Eosinophilic esophagitis, abdominal discomfort, dyspnea, asthma exacerbations, and also generalized pruritus and anaphylaxis have been reported in individual patients [125, 356, 183, 357, 358, 359]. Although the risk for adverse severe systemic reactions with SLIT can be considered lower than with SCIT [125, 348], severe reactions with SLIT have been described in the literature [350, 358]. In some of these cases, however, the treatment did not comply with current standards (non-standardized extracts, rush protocols, excessive doses, patients who had previously discontinued SCIT due to severe reactions) [350]. According to [99], epinephrine was used to treat systemic side effects in 6 patients, but severe anaphylaxis has not been reported here. In the USA, SLIT patients must be prescribed an epinephrine auto-injector for self-treatment of anaphylactic reactions for safety reasons. There is no such recommendation in Europe. As with SCIT, an important risk factor for the occurrence of severe systemic side effects of SLIT is inadequately controlled asthma [350]. 

Regarding the safety profile of SLIT, it is important to remember that most side effects occur at home, with no possibility of rapid medical intervention for (very rare) systemic reactions. It is therefore important to inform patients or their parents about what to do in case of side effects, missed doses, and situations in which SLIT should be temporarily suspended. The latter include elective oral and maxillofacial surgery, the presence of oropharyngeal infections and lesions (ulcers, gingivitis, periodontitis), gastroenteritis, and asthma exacerbations [125]. In the case of local side effects that are distressing for patients, such as pruritus or slight mucosal swelling, the prophylactic intake of an oral H1 receptor antagonist can be considered, especially at the beginning of treatment. In case of repeated stronger local reactions, e.g., angioedema of the lip, the dose should be adjusted, or, if necessary, therapy should be discontinued.

**Recommendation 23:** Because SLIT is performed at home and without immediate medical supervision, the patient should be informed very carefully and comprehensively about the correct use, possible side effects, their management, and risk factors. (*strong consensus, agreement of 100%*)

A history of severe systemic reactions after subcutaneous application of allergens is also a risk factor for potential severe systemic reactions with SLIT [363]. The WAO recommends adopting the SCIT grading of systemic reactions [274] (modified according to [339] (Table 14)) for SLIT and also proposes a uniform classification of local side effects in SLIT (Table 15) [125, 364]. 

The goal of both classifications is to create a simple, standardized reporting system worldwide that allows the frequency and severity of adverse reactions to AIT (SLIT and SCIT) to be more accurately determined [366]. 

Lack of adherence, emerging contraindications, persistent unacceptable local side effects, severe post-treatment reactions, and failure to achieve a clinical response after 1 year of SLIT can be reasons for premature discontinuation of therapy. If SLIT is unsuccessful, the diagnosis ought to be critically reviewed. If no competing allergies can be detected, a change of the preparation can be considered, since the composition of different allergen extracts varies depending on the manufacturer [367].

**Conclusion 39:** Dose-related local adverse reactions in the mouth and throat frequently occur at the beginning of SLIT and carry the risk of early discontinuation of therapy. Systemic reactions, on the other hand, especially severe or anaphylactic ones, have been described in isolated cases, but occur much less frequently than with SCIT.

**Recommendation 24:** Oral H1 antihistamines may be considered as a premedication to reduce distressing local symptoms. (*strong consensus, agreement of 100%*)

### 7.3. Reporting of AIT adverse reactions in Germany, Austria, and Switzerland 

In Germany, the marketing authorization holder has to transmit all of the information on every suspected case of: 1) serious adverse reactions that occur domestically or abroad, within a period of 15 days, 2) non-serious adverse reactions that occur domestically or in a Member State of the European Union, within 90 days after acquiring this knowledge of it, electronically, to the EudraVigilance database pursuant to Article 24 of Regulation (EC) 726/2004. Reporting suspected ADRs in everyday use is of great importance to obtain as much data as possible on the safety of the drugs and to allow continuous monitoring of the risk-benefit ratio of the drugs. Physicians, pharmacists, and other healthcare professionals therefore ought to also report any suspected case of an ADR to the national reporting system (the PEI for allergen preparations used in Germany) (pdf form: send by post, fax or email). In Germany, patients can report ADRs online at https://nebenwirkungen.bund.de. 

In Austria, the Federal Office for Safety in Health Care – BASG is in charge of the responsibilities of public administration. The BASG is directly subordinate to the Austrian Federal Ministry of Health – BMG, representing the owner of AGES, the Republic of Austria. The Austrian Medicines and Medical Devices Agency (AGES MEA) is responsible for assessing the efficacy and safety of medicinal products and medical devices, market surveillance, inspection eg. of manufacturers and clinical trials. When carrying out sovereign activities, the employees of AGES MEA are acting on behalf of the Federal Office for Safety in Health Care – BASG. According to the Austrian Medicinal Products Act and the Pharmacovigilance Ordinance 2013 (https://www.ris.bka.gv.at/GeltendeFassung.wxe?Abfrage=Bundesnormen&Gesetzesnummer=20008606), members of the professional groups of physicians, dentists, veterinarians, midwives, pharmacists, druggists, as well as tradespersons who are authorized to manufacture medicinal products or to engage in wholesale trade in medicinal products pursuant to the Industrial Code 1994, and marketing authorization holders of medicinal specialties are obliged to report any occurring ADRs to the AGES MEA. As in Germany, marketing authorization holders must electronically transmit information on all suspected serious ADRs that have occurred domestically or abroad to the EudraVigilance database within 15 days of becoming aware of them. Information on all suspected non-serious ADRs that have occurred in the European Union must be submitted electronically by the marketing authorization holder to the EudraVigilance database within 90 days of becoming aware of them (AMG Section 75j para. 3). In Austria, patients also have the option of reporting ADRs electronically themselves at https://www.basg.gv.at/marktbeobachtung/meldewesen/nebenwirkungsmeldung-human. 

In Switzerland, since the introduction of the new Medicinal Product Act (Heilmittelgesetz (HMG)) in 2002, healthcare professionals have been subject to a reporting obligation for certain ADRs that are fatal or life-threatening, cause serious or permanent damage, or those that are not or insufficiently mentioned in the drug information (drug compendium) [368]. The report is made using a special form to one of the regional pharmacovigilance centers. These take over the data entry and electronic forwarding (anonymized regarding patient and primary notifier) to Swissmedic. The direct reporting to Swissmedic is electronically at ElViS (Electronic Vigilance System). The reports are edited and estimated in close collaboration with regional pharmacovigilance centers. The latter maintains the central Swiss ADR database and forwards serious and new ADRs to the pharmaceutical companies concerned. It also sends all reports to the World Health Organization (WHO). 

As marketing authorization holders, manufacturers and distributors are also subject to the obligation to report ADRs and quality defects. Notifiable ADRs include serious or previously unknown ADRs, accumulation of known or previously unknown ADRs, quality defects, and unusual restrictions on distribution. 

After marketing authorization in the European Union and Switzerland the marketing authorization holders are obliged to send “period safety update reports” (PSURs) to the PSUR repository, which has been developed by the European Medicines Agency (EMA) in close collaboration with EU Member States and the industry.

**Conclusion 40:** In Germany, Austria, and Switzerland, there is in principle an obligation to report the occurrence of serious AIT ADRs, but there are country-specific regulations regarding the practical implementation.

## 8. Emergency treatment 

Early signs of a severe reaction include burning and itching of palms and soles, perianal or perigenital itching, irritated throat/coughing, urinary and fecal urgency, sneezing attacks, and generalized pruritus. Additional respiratory and/or circulatory symptoms may rapidly occur. Systemic reactions after AIT usually occur within the first 30 minutes after application. When SCIT is used, patients must therefore remain under medical observation for at least 30 minutes after injection and should report any symptoms suspicious of an allergic reaction immediately [33]. However, systemic reactions occurring later than 30 minutes after injection have also been reported [346]. Patients/parents ought to be informed and instructed accordingly. 

Systemic reactions must be treated without delay because of the risk of rapid deterioration [369]. The staff involved must be familiar with the handling of obligatory medications and equipment for allergy emergencies (Table 16) [320]. Treatment of a severe systemic reaction should be appropriate to its stage and in accordance with the anaphylaxis guideline. Appropriate positioning of the patient, intramuscular epinephrine (150 μg for patients with a body weight (BW) of 7.5/15 – 25/30 kg, depending on the preparation; 300 μg for patients > 30 kg BW, 500 μg are possible for BWs > 60 kg), volume therapy via a large-lumen intravenous line, and oxygen (O_2_) administration are among the initial measures for patients with respiratory and/or cardiovascular symptoms. Early use of salbutamol is recommended in cases of obstructive respiratory symptoms. Early intramuscular use of epinephrine for acute therapy of an anaphylactic reaction is recommended to ensure rapid pharmacologic effects (e.g., stabilization of the circulation) [369]. If the response is not sufficient, repeated administration of epinephrine is advisable. For practical reasons, the provision of adequately dosed epinephrine autoinjectors is recommended in order to be able to intervene therapeutically without delay. All staff involved should be regularly trained to take immediate measures in case of systemic allergic reactions [320]. 

Therapeutic recommendations for emergency treatment of anaphylaxis are based on limited data from clinical trials, but they are agreed worldwide (WAO) [369] as well as at the European level (EAACI, [370, 371]) and also nationally with regard to the recommendation of primary use of intramuscular epinephrine [320], which also applies the acute treatment of emergencies during SCIT. The recommendations presented apply analogously to anaphylactic reactions if they would occur during SLIT.

**Conclusion 41:** Risks and consequences of adverse systemic reactions in the setting of AIT can be effectively reduced by staff training, adherence to safety standards, and prompt application of emergency measures, including early administration of intramuscular epinephrine. Details on acute therapy and management of anaphylactic reactions can be found in the corresponding German S2k guideline published in 2021.

**Recommendation 25:** For the performance of AIT and the possible treatment of adverse reactions, extensive staff training, application of appropriate safety standards, and rapid application of necessary emergency measures, including early intramuscular administration of epinephrine should be ensured. (*strong consensus, agreement of 100%*)

## 9. Future perspectives of AIT 

In addition to the well-established administration modes of subcutaneous and sublingual immunotherapy, clinical development of epidermal immunotherapy and oral immunotherapy for the treatment of peanut allergy is well advanced [372, 373]. It can be expected that further allergens and indications will follow in the future. 

Intralymphatic immunotherapy promises advantages, especially with regard to the reduction of necessary injections and the low allergen dose, but needs to be investigated in further studies due to contradictory results [374]. 

The combination of an allergen or allergen mix with TLR agonists (TLR4, TLR9) has been suggested to result in increased immune deviation from Th2 to Th1 and induction of regulatory T cells (Treg) [375]. SCIT preparations containing TLR4 agonists are already on the market, and several positive studies have been performed for combination with TLR9 agonists. 

Concomitant administration of AIT with biologics (anti-IgE, anti-type 2 inflammation [376, 377]) is thought to both reduce side effects and inhibit type 2 allergic inflammation. For the combination of anti-IgE (omalizumab) with AIT, there have been promising results with regard to the reduction of side effects. However, larger, randomized, placebo-controlled trials are needed to transfer this strategy into routine clinical practice [377, 378]. 

No additional clinical benefit has yet been shown for the combination of anti-IL4 with SCIT, although it resulted in suppression of Th2 cells and the late-phase response [376]. However, the marketing authorization and further development of new biologicals that suppress type 2 responses will most likely lead to new insights in this regard. 

Another approach is being pursued by developing allergen-specific passive immunotherapy with human IgG4 monoclonal antibodies to increase the IgG/IgE ratio. A study using IgG4 against Fel d 1 showed a fast-acting and effective reduction of clinical symptoms after provocation with cat allergens [379]. 

The development of modified allergens or of allergen fragments has a great potential in terms of improvement of the effect/side-effect profile, standardization of preparations, and duration of treatment. Strategies range from nucleic acid-based methods and the development of peptides to the recombinant production of wild-type or modified variants of allergens [380]. There are several positive phase II studies for various allergens, but also a negative phase III study for peptides for the treatment of cat allergy patients, the further development of which has been stopped (see Chapter 4.8.2, keyword “*pet allergens”*). 

By applying modern methods of vaccinology, relevant new developments can be expected in this field in the future [9].

**Conclusion 42:** There is a need for further development of allergen immunotherapy in terms of increased and sustained efficacy and safety. Many innovative approaches are currently being developed.

## Summary of conclusions 

The main immune modifications of AIT are i) the temporary induction of regulatory immune cells (DCregs, Tregs, Bregs), ii) the reduction of allergen-specific innate immunity and T helper cell activity, and iii) the formation of allergen-blocking IgG and IgA antibodies. Finally, a “T-cell-normalized” endotype emerges from the primarily Th2-dominated endotype as an immunological prerequisite for clinical allergen tolerance. 

AIT products (SCIT and SLIT) are not comparable due to their heterogeneous composition. Likewise, the allergen concentrations given by different manufacturers to date are also not comparable due to different methods of measuring the active components. For SCIT, non-modified allergens are used as aqueous or physically coupled (semi-depot) extracts, and chemically modified extracts (allergoids) are used as semi-depot extracts. The allergen extracts and allergoids for SLIT are used as aqueous solutions or tablets. In the future, according to the European Pharmacopoeia, it will be mandatory to indicate the quantity of Bet v 1 in birch pollen extracts and Phl p 5a in timothy grass extracts. 

The clinical efficacy of AIT is measured using patient-reported outcomes (PROs) as primary and secondary endpoints. For clinical phase III studies, the EMA stipulates a combined symptom and medication score (CSMS) for the primary outcome parameter. The CONSORT recommendations specify standardized procedures for the evaluation, presentation, and publication of study results. The results of the placebo group are to be described in just as much detail as those of the actively treated group. 

Products containing frequent allergen sources (pollen from sweet grasses (except maize), birch, alder, hazel; house dust mites; bee and wasp venom) must obtain a marketing authorization in Germany according to the Therapy Allergen Ordinance. During the authorization process, quality, efficacy, and safety of these products are assessed. Authorized or otherwise marketable allergen products demonstrating a positive risk-benefit ratio according to EMA guidelines should preferably be applied. Named-patient products are used to prescribe rare allergen sources for AIT. They cannot be mixed with the allergens listed in the Therapy Allergen Ordinance. Country-specific regulations apply to Austria and Switzerland. 

Allergic rhinoconjunctivitis and allergic bronchial asthma cause considerable direct, indirect, and intangible costs for society as a whole. AIT is significantly more cost-effective over the long term when indicated and used in accordance with guidelines compared to pharmacotherapy alone provided that adherence to therapy is good. The choice of the AIT product has to be decided on an individual basis, whereby clinical benefits are given priority over costs according to German social law. 

Systematic reviews and meta-analyses demonstrate the efficacy of SCIT and SLIT for certain indications, allergens, and age groups. The data from the controlled studies differ significantly in terms of their scope, quality, preparations, and dosing regimens and require a product-specific evaluation. A broad transfer of the efficacy of certain preparations to all preparations administered in the same way is not endorsed. 

A product-specific evaluation of the individual AIT preparations according to clearly defined criteria is recommended. On the DGAKI website (https://dgaki.de/leitlinien/s2k-leitlinie-ait/) a tabular overview with AIT product-specific information is given, which includes the homologous groups of grass, tree pollen (Betulaceae), and house dust mite allergen preparations distributed in Germany and/or Austria and/or Switzerland. 

The efficacy of SCIT in ARC in grass pollen allergy has been demonstrated very well by numerous studies in adult patients; in children and adolescents, this has been proven by few studies. An uncontrolled trial and an RWE study showed asthma-preventive effects in children and adolescents. In general, there are product-specific differences in the documentation of clinical efficacy which underlines the importance of a product-specific evaluation. This also applies to all of the following allergen groups. 

The efficacy and safety of SLIT in ARC caused by grass pollen allergy in adults and children is very well documented. However, product-specific differences exist. A controlled study has indicated asthma-preventive effects in children and adolescents. 

The efficacy of SCIT with grass pollen extracts in seasonal allergic asthma caused by grass pollen allergy has been proven well in adult patients and has been proven in children only in a few studies. 

There are only very few representative studies on the efficacy of SLIT in adults with seasonal bronchial asthma induced by grass pollen allergy, and few representative studies in the age groups of children and adolescents. Based on the current data, there is only limited evidence to recommend SLIT in allergic asthma due to grass pollen. 

The efficacy of SCIT in ARC caused by tree pollen (Betulaceae-) allergy in adults has been well documented by numerous studies, whereas there is a lack of specific studies in children and adolescents. First real-world analysis data based on health insurance prescription are indicating efficacy in all age groups. 

The safety and efficacy of SLIT in ARC induced by tree pollen allergy are very well documented in adult patients. For children and adolescents with tree pollen allergy, a few studies indicate that the treatment is safe and efficacious. 

A DBPC trial indicates that SCIT with tree pollen extracts is effective in adults with seasonal allergic asthma caused by tree pollen (Betulaceae) allergy. 

The efficacy of SCIT with tree pollen extracts in adults and in children with seasonal allergic asthma has been investigated only to a low extent. However, data from real-world analysis based on health insurance prescription data suggest efficacy in this indication. 

SLIT with tree pollen can be safely applied in patients with controlled asthma. However, the studies on ARC showed divergent effects on asthma symptom control. 

For other tree pollen not cross-reactive to the birch and beech families, such as ash, cypress, and plane tree pollen, little evidence is available for clinical efficacy, and the availability of therapeutic extracts is limited. 

Data are available on the safety and efficacy of SCIT using house dust mite extracts in ARC for adult patients. The safety and efficacy of this treatment in children has been demonstrated in studies with small numbers of cases, but a lack of double-blind, placebo-controlled SCIT studies following modern methodological standards remains. 

For some house dust mite SLIT preparations, the efficacy and safety in ARC in adult and adolescent patients have been proven in studies with large numbers of cases. Studies have also shown the efficacy and safety of SLIT in children with ARC and house dust mite allergy. 

For adults, the efficacy and safety of SCIT for allergic asthma caused by house dust mite allergy has been proven in studies. However, the clinical endpoints used in the studies differ significantly and therefore a comparison of the effect sizes as demonstrated in different studies is not possible. The data for children and adolescents are limited, although evidence exists for efficacy and safety of house dust mite SCIT in allergic asthma in this age group. 

For adults, efficacy and safety of SLIT for allergic asthma caused by house dust mite allergy has been proven in studies. However, the clinical endpoints used in the studies differ significantly and therefore a comparison of the effect sizes as demonstrated in different studies is not possible. The data for children and adolescents are limited, although evidence exists for efficacy and safety of house dust mite SLIT in allergic asthma in this age group. 

Regarding preparations with storage mite extracts, only very little evidence for its clinical efficacy is available, and the availability of preparations for AIT is limited. 

Efficacy of AIT in ragweed pollen allergy is well established for SCIT in adults with ARC and weakly established in asthma; data for children are lacking. For SLIT, the efficacy of a tablet preparation containing a ragweed pollen extract is very well demonstrated for adults and children with ARC. 

For the efficacy of AIT with other allergen extracts (except grasses, birch, mites, ragweed), a few studies for SCIT and SLIT are available but currently do not allow an evaluation of therapy efficacy. 

Various routes of application of AIT have been studied in peanut allergy, with the most clinical trials being available for oral tolerance induction (OTI). One OTI preparation has gained market authorization for the treatment of children and adolescents with a confirmed diagnosis of peanut allergy. 

No AIT preparation has gained market authorization for the treatment of AD. However, AD is not a contraindication to AIT if indicated otherwise. In most studies, SCIT and SLIT with house dust mite extracts have demonstrated positive effects in patients with AD related to house dust mite allergy. 

Various prerequisites are essential for the indication to initiate AIT. Under certain conditions, AIT can also be used for patients with milder symptoms with the treatment goal of disease modification. 

Clear communication with the patient and family members regarding the application route and organizational precautions is essential for determining the therapy. 

Component resolved diagnostics can be helpful in estimating the probability of success of AIT, particularly in the case of polysensitization. 

Various contraindications are found for AIT, with only very minor differences between the application routes. 

When establishing the indication, factors that may impact the clinical efficacy must be taken into account. If there is an indication, and after considering comorbidities, AIT is also possible in patients over 65 years of age or in patients with autoimmune disease. Differences between SCIT and SLIT are primarily to be considered with regard to contraindications. Even if contraindications are present, there may be an indication for AIT in justified individual cases. 

Pharmacotherapy is the basis of therapy for both ARC and asthma, and AIT is applied in addition to it. 

In Germany, Austria, and Switzerland, different legal provisions apply to physicians applying AIT. 

A detailed and well-documented patient information is a prerequisite for the implementation of AIT. The documents “Treatment Information Sheet SCIT” and “Treatment Information Sheet SLIT” can be helpful in this regard. 

Irrespective of the mode of administration, therapy adherence of AIT patients is lower than assumed by physicians, although it is of decisive importance for treatment success. Improving adherence to AIT is one of the most important tasks to ensure optimal effectiveness of the treatment. Consideration of patient-specific requirements, comprehensive instruction of the patients, and optimized practice/clinic management are decisive for a high degree of adherence. 

Various precautions and procedures are helpful to ensure that SCIT is performed in accordance with the guidelines. These can also be found in the summary of product characteristics and package leaflet of each SCIT preparation. 

Various precautions and procedures are helpful to ensure that SLIT is performed in accordance with the guidelines. These are based on the recommendations in the summary of product characteristics and package leaflet of the SLIT preparations. 

Based on current knowledge, the use of aluminium-adjuvanted SCIT preparations does not pose a risk of toxic effects in children or adults. 

Most adverse reactions are mild to moderate in severity and are easy to manage. The occurrence of severe, potentially life-threatening systemic reactions in SCIT is possible but very rare if all safety measures are observed. 

Dose-related local adverse reactions in the mouth and throat frequently occur at the beginning of SLIT and carry the risk of early discontinuation of therapy. Systemic reactions, on the other hand, especially severe or anaphylactic ones, have been described in isolated cases, but occur much less frequently than with SCIT. 

In Germany, Austria, and Switzerland, there is in principle an obligation to report the occurrence of serious AIT ADRs, but there are country-specific regulations regarding the practical implementation. 

Risks and consequences of adverse systemic reactions in the setting of AIT can be effectively reduced by staff training, adherence to safety standards, and prompt application of emergency measures, including early administration of intramuscular epinephrine. Details on acute therapy and management of anaphylactic reactions can be found in the corresponding German S2k guideline published in 2021. 

There is a need for further development of allergen immunotherapy in terms of increased and sustained efficacy and safety. Many innovative approaches are currently being developed. 

## Acknowledgment 

The authors are thankful to Veronika Luger (Dustri, Germany) for translation and Isabel Hyde (Leeds Institute of Rheumatic and Musculoskeletal Medicine (LIRMM), University of Leeds, Leeds, United Kingdom) for native-speaker checking the translated (English) manuscript. 

## Funding 

The consensus conference in Hannover (premises, technical equipment, dinner, lunch) as well as the digital conferences (GoToTraining) and the AWMF moderator were financed by the DGAKI, as were the methodologists for the evidence report. The travel and accommodation costs for the 1st consensus conference in the presence of the participants were financed by the respective specialist societies or organizations. In addition, no compensation was paid. 

## Conflict of interest 

The conflicts of interest were recorded using the AWMF portal interessenerklaerung-online.de, evaluated by the conflict of interest officer of the DGAKI (for details see the guideline report) and tabulated in accordance with the AWMF. The guideline report and conflict of interest table are available at www.awmf.org/leitlinien/detail/ll/061-004.html. 

**Table 1.** Important terms of the German Medicinal Products Act (AMG) (https://www.gesetze-im-internet.de/englisch_amg/englisch_amg.html) and special features in Austria and Switzerland, partially translated by the authors.

**Finished medicinal products**
Section 4, subsection 1 AMG: “Finished medicinal products are medicinal products that are manufactured beforehand and placed on the market in packaging intended for distribution to the consumer, or other medicinal products intended for distribution to the consumer in the preparation of which any form of industrial process is used, or medicinal products that are produced commercially, except in pharmacies….”
**Marketing authorization**
Section 21, subsection 1 AMG: “Finished medicinal products that are medicinal products as defined in section 2 (1) or subsection (2) no. 1, may only be placed on the market within the purview of this Act, if they have been authorized by the competent higher federal authority …”
**Custom formulations**
Section 21, subsection 2 AMG: “A marketing authorization (Zulassung) is not required for medicinal products that … 1g. are therapeutic allergens manufactured to order for individual patients”
**Important terms of the Austrian Medicinals Products Act** ^1^
§ 7a (1): Medicinal products that contain antigens or semi-antigens and are used for the identification of specific defense and protective substances, or used for desensitization or hyposensitization may, unless they are invariably prepared in advance with the same composition or are placed on the market for sale to the consumer or user in a specific form under the same name, only be dispensed in Austria or made available for dispensing in Austria if the Federal Office for Safety in Health Care has approved by means of a notification the manufacturing process to be used for this medicinal product, including the chemical-pharmaceutical documentation.
**Situation in Switzerland** ^1^
According to the Swiss Federal Act on Medicinal Products and Medical Devices of December 15, 2000 (Heilmittelgesetz (HwMG), Art. 9, subsection. 1), allergen preparations for AIT are classified as medicinal products that require authorization (SR812.21, https://www.fedlex.admin.ch/eli/cc/2001/422/en). Allergen preparations that are used in accordance with the exemption clause (Art. 9, subsection 2 HMG), e.g., as magistral formula (patient-specific mixtures of allergens), are exempt from authorization. In 2010, a new ordinance came into force for the simplified authorization of allergen preparations (Allergen Ordinance, AllergV SR812.216.2, https://www.fedlex.admin.ch/eli/cc/2010/61/de). The simplification of the authorization procedure consists in the fact that the documentation for the authorization can be based on published literature (from scientifically recognized sources) or on documents from another allergen product (reference product from the same manufacturer). Allergen preparations that contain recombinantly produced allergens or genetically modified organisms are excluded from the simplified authorization procedure. Accordance to Art. 13 of the HMG, if an authorization for allergen preparations already exists in a country with comparable drug controls and a comparable authorization procedure, the results can also be taken into account for the authorization in Switzerland.

^1^Translated by authors.

**Table 2.** List of therapy allergens requiring marketing authorization in Germany* [20].

Species of the family Poaceae except Zea mays^1^ (sweet grasses except maize)
*Betula sp.* (species of the genus birch)
*Alnus sp.* (species of the genus alder)
*Corylus sp.* (species of the genus hazel)
*Dermatophagoides sp.* (species of the genus house dust mite)
Bee venom
Wasp venom

*List of therapy allergens that require a marketing authorization according to the German Therapy Allergen Ordinance [20]. After the expiration of the transitional provisions, they may not be placed on the market neither as single allergen source preparations nor in mixtures without marketing authorization. ^1^Note by the authors: In the original text of the TAO there has been given “Poa mays” by mistake, correct is “Zea mays”.

**Table 3.** Marketing authorization procedures for medicinal products in the European Union (EU).

**National procedure** through which the medicinal product is authorized only in the respective member state.
**Mutual recognition procedure** if the preparation is already authorized in a member state of the EU and the authorization is to be extended to further member states.
**Decentralized procedure** if the medicinal product does not yet have a national marketing authorization and is to be authorized in parallel in several EU member states.
**Centralized procedure** (simultaneous marketing authorization in all EU member states), which must be applied when it comes to medicinal products that are named in the appendix to EC Regulation 726/2004 (e.g., medicinal products manufactured using biotechnological processes); it can also be used for other medicinal products under certain conditions. These are coordinated by the European Medicines Agency (EMA).

**Table 4.** Examples of NPPs for AIT with allergen groups not regulated by TAO* [20].

Mugwort pollen (*Artemisia vulgaris*)
Ash pollen (*Fraxinus excelsior*)
Alternaria (*Alternaria alternata*)
Animal allergens, e.g., cat (*Felis domesticus*)
Storage mites (e.g., *Acarus siro*)

*Not as an admixture with the allergen groups of the TAO (Table 2, [20]), otherwise they would be subject to the Regulation.

**Table 5.** Current indication-related meta-analyses (MA) and systematic reviews (SR) on AIT.

**Indication** **(type of study)**	**Number of included studies**	**Verbatim quote of the conclusion of the review article as given in the abstract**	**Year of publication [reference]**
Allergic rhinoconjunctivitis (MA)	160	*“AIT is effective in improving symptom, medication, and combined symptom and medication scores in patients with allergic rhinoconjunctivitis while on treatment, and there is some evidence suggesting that these benefits are maintained in relation to symptom scores after discontinuation of therapy.”*	2017 [98]
Allergic rhinoconjunctivitis (SR)	17	*“We found moderate-to-strong evidence that SCIT and SLIT can, in appropriately selected patients, reduce symptoms and medication requirements in patients with allergic rhinoconjunctivitis with reassuring safety data. This evidence does however need to be interpreted with caution, particularly given the heterogeneity in the populations, allergens and protocols studied. There is a lack of data on the relative effectiveness, cost-effectiveness and safety of SCIT and SLIT.”*	2017 [99]
Allergic asthma (MA)	98	*“AIT can achieve substantial reductions in short-term symptom and medication scores in allergic asthma. It was however associated with a modest increased risk of systemic and local adverse events. More data are needed in relation to secondary outcomes, longer-term effectiveness and cost-effectiveness”*	2017 [100]
Allergic asthma (SR)	9	*“AIT has the potential to achieve reductions in symptom and medication scores, but there is no clear or consistent evidence that measures of lung function can be improved. Bearing in mind the limitations of synthesizing evidence from systematic reviews and the fact that these reviews include mainly dated studies, a systematic review of current primary studies is now needed to update this evidence base, estimate the effectiveness of AIT on asthma outcomes and to investigate the relative effectiveness, cost-effectiveness and safety of SCIT and SLIT.”*	2017 [101]
Food allergy (MA)	31	*“AIT may be effective in raising the threshold of reactivity to a range of foods in children with IgE-mediated food allergy whilst receiving (i.e. desensitization) and post-discontinuation of AIT. It is, however, associated with a modest increased risk in serious systemic adverse reactions and a substantial increase in minor local adverse reactions. More data are needed in relation to adults, long term effects, the impact on QoL and the cost-effectiveness of AIT”*	2017 [102]
Peanut allergy (MA, SR)	12	*“In patients with peanut allergy, high-certainty evidence shows that available peanut oral immunotherapy regimens considerably increase allergic and anaphylactic reactions over avoidance or placebo, despite effectively inducing desensitisation. Safer peanut allergy treatment approaches and rigorous randomised controlled trials that evaluate patient-important outcomes are needed. ”*	2019 [103]
Insect venom allergy (MA, SR)	17	*“The limited available evidence suggested that VIT is effective in reducing severe subsequent systemic sting reactions and in improving disease-specific quality of life. VIT proved to be safe and no fatalities were recorded in the studies included in this review. The cost-effectiveness of VIT needs to be established.”*	2017 [104]
Atopic dermatitis (MA)	12	*“We found no consistent evidence that SIT is effective for treating atopic eczema, but due to the low quality of evidence further research is needed to establish whether SIT has a role in atopic eczema treatment.”*	2016 [105]
Allergic asthma, SLIT (MA)	66	*“Despite continued study in the field, the evidence for important outcomes such as exacerbations and quality of life remains too limited to draw clinically useful conclusions about the efficacy of SLIT for people with asthma. Trials mostly recruited mixed populations with mild and intermittent asthma and/or rhinitis and focused on non-validated symptom and medication scores. The review findings suggest that SLIT may be a safe option for people with well-controlled mild-to-moderate asthma and rhinitis who are likely to be at low risk of serious harm, but the role of SLIT for people with uncontrolled asthma requires further evaluation.“*	2020 [106]

**Table 6.** Indication: Prerequisites for the use of AIT.

1) Moderate to severe intermittent and persistent allergic rhinitis/rhinoconjunctivitis and/or at least partially controlled allergic asthma*,
and 2) evidence of a corresponding clinically relevant allergic sensitization (Figure 3),
and 3) symptoms despite symptomatic therapy and/or allergen avoidance,
and 4) evidence of efficacy of the planned AIT for the respective indication and age group

*Also in the case of milder symptoms with the treatment goal of a disease-modifying effect of AIT and if 2) and 4) are present.

**Table 7.** Criteria for examining the individual suitability of patients for the respective AIT application route, modified from [33].

	**Pros**	**Cons**
**Subcutaneous route**	Application by doctor = certainty about administration	Regular visits to the doctor (time-consuming)
	Frequent doctor-patient contact > regular monitoring of the course of the AIT, side effects, and underlying disease(s) of the patients possible	Possible fear of injections
		At least 30 minutes of post-injection monitoring time
		Risk of systemic allergic reactions *(very rare)*
		Risk of local side effects *(frequent)*
**Sublingual route**	Non-painful procedure	Risk of local side effects *(very frequent, mostly mild and self-limiting)*
	Can be carried out at home (usually first application at the doctor’s office with 30-minute monitoring)	Usually daily application necessary over a longer period of time (pre-/co-seasonal, i.e., several months, or perennial > daily “remembering”)
	Visits to the doctor’s office rarely necessary	Mucosal contact for 2 minutes and motivation of the patients necessary (check especially with children)
	Very low risk of systemic reactions (lower than with SCIT)	

**Table 8.** Important points to discuss with the patient/family when establishing the indication for AIT and selecting of the application route and AIT product.

+ Chance of success and safety
+ Availability of a therapy allergen with documented efficacy (evidence)
+ Costs
+ Need for adherence and persistence (for SCIT and SLIT)
+ Individual peculiarities (e.g., needle phobia, time in everyday life required for therapy, longer absences, sporting activity, adherence to regular application of medication in general)
+ Procedure (frequent visits to the doctor, waiting times, travels to the doctor’s office, opening hours of the practice/outpatient clinic), prescription requirements
+ Accessibility and qualification of the practice/ambulance for
–SCIT: distance of practice/outpatient clinic to home, doctor with training in allergy, trained practice team, flexible office hours
–SLIT: flexible prescription availability, team is trained for consultation about side effects, questions about therapy, etc.

NOTE: A reliable doctor-patient relationship with clear communication, thorough consideration, and discussion of the above criteria and finally individual selection of the therapy allergen increases the chances of good long-term adherence and persistence over 3 (– 5) years.

**Table 9.** Helpful allergen components when establishing the indication for AIT with inhaled allergens (major allergens^a^ versus panallergens^b^).

Major allergens^a^
Bet v 1 → Birch, *Betula pendula* (formerly *Betula verrucosa*) is the most allergenic representative of the *Betulaceae* family, which are generally very strongly cross-reactive (particularly alder, hazelnut pollen, weaker to oak and beech)
Phl p 1/5 → grasses, *Phleum pratense* (timothy grass)
Der p 1/2 → House dust mites, *Dermatophagoides pteronyssinus,* very high cross-reactivity with *Dermatophagoides farinae*
Alt a 1 → Alternaria, *Alternaria alternata*
Ole e 1 → Ash: *Fraxinus excelsior* – no commercially available components, the very high cross-reactivity allows testing with the major allergen from the olive tree: *Olea europaea*
Art v 1 → Mugwort, *Artemisia vulgaris*
Amb a 1 → Ragweed, *Ambrosia artemisiifolia*
Pla l 1 → Ribwort plantain, *Plantago lanceolata*
Components that explain positive skin tests but are not valid for establishing the indication for immunotherapy (panallergens^b^)
Profilins^c^: e.g.: Amb a 8 (ragweed), Ara h 5 (peanut), Bet v 2 (birch), Cor a 2 (hazelnut),
Hev b 8 (latex), Phl p 12 (grass), Tri a 12 (wheat^b^)
Polcalcins^c^: e.g.: Aln g 4 (alder), Amb a 9 (ragweed), Art v 5 (mugwort), Bet v 4 (birch), Phl p 7 (grass)

^a^An allergen is considered a major allergen if it leads to an IgE sensitization in more than 50% of the affected allergic subjects. The name of an allergen component is derived from the first three letters of the genus name and the first letter of the species name, e.g., timothy grass *Phleum pratense* → Phl p 1. The numbering often follows the order of the first description, and thus, unfortunately the same numbers do not automatically mean cross-reactivity. The cross-reactivities of some allergen families are so high that it is not necessary to determine the individual components separately: *Betulaceae* (PR-10 proteins): Bet v 1 (birch) ←→ Aln a 1 (alder) ←→ Cor a 1 (hazelnut); Grasses (grasses group 1 allergen): Phl p 1 (timothy grass) ←→ Lol p 1 (ryegrass) ←→ Tri a 1 (wheat); House dust and flour mites: cysteine proteases: Der p 1 ←→ Der f 1, NPC2 family: Der p 2 ←→ Der f 2. ^b^Panallergens are found in many species and are generally clinically insignificant, but nevertheless explain irrelevant positive extract-based skin and/or blood tests: e.g., profilins are currently described from 48 plant species. The daily updated international WHO/IUIS online directory for all allergen components can be accessed at www.allergen.org. ^c^One candidate each (Bet v2 or Phl p12 or Bet v4 or Phl p7) is sufficient to demonstrate sensitization to profilins and polcalcins.

**Table 10.** Contraindications for AIT.

Contraindications^1,4^	
SCIT	SLIT
Uncontrolled asthma^2^	
Severe systemic reactions (grades 4 and 5 according to WAO definitions [274]) in past AIT procedures	
Malignant neoplastic diseases with current clinical significance	
Severe systemic autoimmune diseases, immunodeficiencies, relevant immunosuppression (due to possible limited immunological effectiveness of AIT)^3^	
Insufficient adherence, severe psychiatric disorders	
Untreated, chronic infection (e.g., HIV, hepatitis C)	
	History of inflammatory gastrointestinal diseases (e.g., eosinophilic esophagitis), acute and chronic recurrent diseases and open wounds of the oral cavity.

^1^In justified individual cases, AIT is always possible after weighing the benefits and risks, even if the contraindications mentioned are present. ^2^After modifying an uncontrolled into a partially controlled or controlled asthma through optimization of the anti-asthma therapy, AIT is possible in principle. ^3^The following organ-related autoimmune diseases do not represent a contraindication for AIT: Hashimoto’s thyroiditis, rheumatoid arthritis (except for systemic form, Still’s disease), ulcerative colitis and Crohn’s disease, type 1 diabetes mellitus. ^4^When assessing the contraindications, the preparation-specific Summary of Product Characteristics (SmPC) and package leaflet must be taken into account. Performing AIT before the age of 5 years is possible in individual cases but is considered off-label use (Exception: peanut OTI, which is also approved for children under 5 years of age).

**Table 11.** Measures to increase therapy adherence to AIT (modified from [33, 305, 307, 308, 309, 319]).

A. Patient-specific requirements	Informed patient
	Corresponding disease burden and perception
	Compatibility of the planned AIT with the patient’s everyday life (time, routine processes, regular doctor follow-up)
B. Patient information and instruction	Accepting the need for therapy
	Knowing about the effectiveness of AIT with appropriate adherence
	Realistic expectations of AIT
	Confidence in the safety of the therapy
C. Management in practice and clinic	Extensive experience with the corresponding route of administration and the AIT products used
	Contact person for any questions and uncertainties of the patients
	Recall systems
	Good practice/clinic management (making of appointments, sufficient number of staff)
	Resources for comprehensive patient information and guideline-compliant implementation of AIT

**Table 12.** Recommendable procedures and precautions when performing SCIT.

1. Check whether the planned injection interval has been met and ask the patient about their general condition, medication, current allergic symptoms, and tolerability of the previous injection in order to rule out contraindications and to individually adjust the dose to be administered if necessary.
2. Document the injection date and the dose to be administered in the patient documentation (e.g., in the injection protocol of the package leaflet).
3. Take out the appropriate vial. Check patient data, allergen composition, concentration, and bottle expiry date.
4. Swirl the bottle gently to mix the contents evenly. Avoid formation of foam and do not use the preparation if it is or was frozen.
5. Disinfect the sealing plug with an alcohol swab and withdraw the appropriate volume with a sterile 1-mL disposable syringe.
6. After cleaning and disinfecting the injection site, lift a skin fold a hand’s breadth above the elbow, aspirate (multiple times if necessary), and then inject slowly and strictly subcutaneously.
7. Monitor the patient for at least 30 minutes after the injection. If adverse events occur during this period, observation must be extended until the state of health returns to normal. Anaphylactic reactions must be treated promptly according to the AWMF anaphylaxis guideline [320] and may require hospitalization.
8. Explain to the patient that if signs of a side effect appear during the observation period or later, he/she has to contact the treating physician or their representative immediately. If necessary, give the patient medication to treat late-onset reactions.

**Table 13.** Risk factors for systemic reactions during AIT (modified from [328, 330, 344, 349, 350, 351]).

Uncontrolled, inadequately treated asthma
Augmentation factors such as physical exertion
Current allergic symptoms
Acute infections
Mast cell disease and elevated tryptase
High degree of sensitization of the patient
Previous anaphylactic reactions during AIT
Inadequate dose escalation during initiation therapy and dosing errors in general
Unsuitable injection technique (for SCIT)

**Table 14.** Grading of systemic adverse events in allergen immunotherapy according to the 2017 World Allergy Organization (WAO) criteria (from [274] (modified based on [339])).

Grade 1	Grade 2	Grade 3	Grade 4	Grade 5
			Anaphylaxis	
Symptom(s)/signs(s) from 1 organ system present	Symptom(s)/sign(s) from ≥ 2 organ symptoms listed in grade 1	Lower airway – Mild bronchospasm, e.g., cough, wheezing, shortness of breath which responds to treatment	Lower airway – Severe bronchospasm, e.g., not responding or worsening in spite of treatment	Lower or upper airways – Respiratory failure^§^
Cutaneous – Urticaria and/or erythema-warmth and/or pruritus, other than localized at the injection site And/or – Tingling, or itching of the lips* or – Angioedema (not laryngeal)*				
OR		AND/OR	AND/OR	AND/OR
Upper airways – Nasal symptoms (e.g., sneezing, rhinorrea, nasal pruritus, and/or nasal congestion) And/or – Throat-clearing (itchy throat)* And/or – Cough not related to bronchospasm		Gastrointestinal – Abdominal cramps* and/or vomiting/diarrhea – Any symptom(s)/sign(s) from grade 1 would be included	Upper airway – Laryngeal edema with stridor – Any symptom(s)/ sign(s) from grades 1 or 3 would be included	Cardiovascular – Collapse/hypotension^†§^ And/or – Loss of consciousness (vasovagal excluded) – Any symptom(s)/ sign(s) from grades 1, 3, or 4 would be included
OR		OTHER		
Conjunctival – Erythema, pruritus, or tearing		– Uterine cramps		
OR				
Other symptoms – Nausea – Metallic taste				

Taken from [274]: The final grade of the reaction is not determined until the event is over, regardless of the medication administered to treat the reaction. The final report should include the first symptom(s)/sign(s) and the time of onset after the causative agent exposure and a suffix reflecting if and when epinephrine was or was not administered: a, ≤ 5 minutes; b, > 5 minutes to ≤ 10 minutes; c, > 10 to ≤ 20 minutes; d, > 20 minutes; z, epinephrine not administered. Final report: Grade 1 – 5; a-d, or z; First symptom(s)/sign(s); Time of onset of first symptom(s)/signs(s). (Case example in [274]). *Application-site reactions would be considered local reactions. Oral mucosa symptoms, such as pruritus, after SLIT administration, or warmth and/or pruritus at a subcutaneous immunotherapy injection site would be considered a local reaction. However, tingling or itching of the lips or mouth could be interpreted as a SAR if the known allergen, eg, peanut, is inadvertently placed into the mouth or ingested in a subject with a history of a peanut-induced SAR. Gastrointestinal tract reactions after SLIT or oral immunotherapy (OIT) would also be considered local reactions, unless they occur with other systemic manifestations. SLIT or OIT reactions associated with gastrointestinal tract and other systemic manifestations would be classified as SARs. SLIT local reactions would be classified according to the WAO grading system for SLIT local reactions (364). A fatal reaction would not be classified in this grading system but rather reported as a serious adverse event. ^†^Hypotension is defined per the National Institute of Allergy and Infectious Disease/Food Allergy and Anaphylaxis Network Expert Panel criteria [365]:“*Reduced blood pressure after exposure to known allergen for that subject (minutes to several hours). *A) Infants and children: low systolic blood pressure (age-specific) or greater than 30% decrease in systolic blood pressure. Low systolic blood pressure for children is defined as follows: – 1 month to 1 year: < 70 mmHg; – 1 – 10 years: < 70 mmHg + (2 × age); – 11 – 17 years: < 90 mmHg; B) Adults: systolic blood pressure of less than 90 mmHg or greater than 30% decrease from that person’s baseline. ^§^Death would be reported as a serious adverse event (*added by authors*).

**Table 15.** Grading of local adverse events in sublingual immunotherapy (SLIT) according to the 2013 World Allergy Organization (WAO) criteria (from [364]).

Symptom/clinical sign	Grade 1: Mild	Grade 2: Moderate	Grade 3: Severe	Unclear severity
Pruritus/swelling of mouth, tongue, or lip; throat irritation*, nausea, abdominal pain, vomiting, diarrhea, heartburn, or uvular edema	– Not troublesome *and * – no symptomatic treatment required *and * – no discontinuation of SLIT because of local side effects	– Troublesome *or * – Requires symptomatic treatment *and * – No discontinuation of SLIT because of local side effects	Grade 2 *and * SLIT discontinued because of local side effects	Treatment is discontinued, but there is no subjective, objective, or both description of severity from the patient/physician.

Each local AE can be early (< 30 minutes) or delayed. *E.g., palate itching, burning or swelling in the throat (added by guideline authors.

**Table 16.** Emergency equipment for the treatment of anaphylactic reactions in AIT (adapted from [320]).

Stethoscope
Blood pressure monitor
Pulse oximeter, possibly blood glucose meter
Tourniquet, venous catheters (in different sizes), syringes, infusion set, adhesive tape for catheter fixation
Oxygen and nebulizer set with oxygen mask (various sizes)
Resuscitator bag with masks (different sizes)
Suction device
Guedel tube, where appropriate
Volume (e.g., balanced full electrolyte solution)
Drugs for injection: Epinephrine, glucocorticoid, H1 receptor antagonist
Short-acting β2-adrenoceptor agonist, e.g., salbutamol for inhalation (preferably as a metered dose aerosol with an adequate inhalation aid)

**Abbreviations TableAbbreviations:** Abbreviations.

AAAAI	American Academy of Allergy, Asthma and Immunology
ACAAI	American College of Asthma, Allergy and Immunology
AD	Atopic dermatitis
ADR	Adverse drug reaction
AeDA	Medical Association of German Allergologists (Ärzteverband Deutscher Allergologen)
AGES MEA	Austrian Medicines and Medical Devices Agency
AIT	Allergen immunotherapy
Al(OH)3	Aluminium hydroxide
AMG	Medicinal Products Act (Arzneimittelgesetz)
ARC	Allergic rhinoconjunctivitis
AWMF	Association of the Scientific Medical Societies in Germany (Arbeitsgemeinschaft der Wissenschaftlichen Medizinischen Fachgesellschaften)
BASG	Federal Office for Safety in Healthcare (Bundesamt für Sicherheit im Gesundheitswesen),
BdP	Federal Association of Pneumologists, Sleep and Respiratory Physicians (Bundesverband der Pneumologen)
Bregs	Regulatory B cells
BVDD	Professional Association of German Dermatologists (Berufsverband der Deutschen Dermatologen)
BVHNO	German Professional Association of Otolaryngologists (Berufsverband der Hals-Nasen-Ohrenärzte)
BVKJ	German Association of Pediatric and Adolescent Care Specialists (Berufsverband der Kinder- und Jugendärzte)
BW	Body weight
cART	Combined antiretroviral therapy
CONSORT	Consolidated standards of reporting trials
CRD	Component resolved diagnostics
CSMS	Combined symptom and medication score
DAAB	German Allergy and Asthma Association (Deutscher Allergie- und Asthmabund)
DBPC	Double-blind placebo-controlled
DCs	Dendritic cells
DDG	German Dermatological Society (Deutsche Dermatologische Gesellschaft)
DELBI	German Instrument for Methodological Guideline Appraisal (Deutsches Leitlinien-Bewertungsinstrument)
DGAKI	German Society for Allergology and Clinical Immunology (Deutsche Gesellschaft für Allergologie und klinische Immunologie)
DGHNO-KHC	German Society of Oto-Rhino-Laryngology, Head and Neck Surgery (Deutsche Gesellschaft für Hals- Nasen-Ohren-Heilkunde, Kopf- und Hals-Chirurgie)
DGKJ	German Society of Pediatrics and Adolescent Medicine (Deutsche Gesellschaft für Kinder- und Jugendmedizin)
DGP	German Respiratory Society (Deutsche Gesellschaft für Pneumologie und Beatmungsmedizin)
EAACI	European Academy of Allergy and Clinical Immunology
EASSI	European Survey on Adverse Systemic Reactions in Allergen Immunotherapy
ElViS	Electronic Vigilance System
EMA	European medicines agency
EU	European union
FAS	Full analysis set
GCP	Good clinical practice
GINA	Global Initiative for Asthma
GMP	Good manufacturing practice
GPA	Society of Pediatric Allergology and Environmental Medicine (Gesellschaft für Pädiatrische Allergologie und Umweltmedizin)
GPP	Society of Pediatric Pulmonology (Gesellschaft für Pädiatrische Pneumologie)
H1	Histamin 1
HIV	Human immunodeficiency virus
HMG	Heilmittelgesetz
HR	Hazard ratio
HRQL	Health-related quality of life
ICER	Incremental cost-effectiveness ratio
IFN	Interferon
IL	Interleukin
ILC2	Innate lymphoid cells 2
ITT	Intention-to-treat
LABA	Long-acting β-2-agonists
LAMA	Long-acting muscarinic receptor antagonists
LTRA	Leukotriene receptor antagonists
MASK-air	Mobile Airways Sentinel Network
MCs	Mast cells
NPP	Named patient products
ÖGAI	Austrian Society for Allergy and Immunology (Österreichische Gesellschaft für Allergologie und Immunologie)
OTI	Oral tolerance induction
PBTK	Physiologically based toxicokinetics
PEI	Paul-Ehrlich-Institut
PhVO	Pharmacovigilance Ordinance
PP	Per-protocol
PPP	Peak Pollen Period
PRISMA	Preferred Reporting Items for Systematic Reviews and Meta-Analyses
PROs	Patient-reported outcomes
PSURs	Periodic Safety Update Reports
QALI	Quality-adjusted life year
RWE	Real-world evidence
SAR	Seasonal allergic rhinoconjunctivitis
SCIT	Subcutaneous immunotherapy
SCORAD	Scoring atopic dermatitis
SGAI	Swiss Society for Allergology and Immunology (Schweizerische Gesellschaft für Allergologie und Immunologie)
SLIT	Sublingual immunotherapy
SMD	Standard mean difference
SmPC	Summary of Product Characteristics
TAO	Therapy Allergen Ordinance
TCS	Total combined score
Treg	Regulatory T cells
TSS	Total symptom score
VAS	Visual analogue scales
WAO	World Allergy Organization
WHO	World Health Organization
